# Borderline *rpoB* mutations transmit at the same rate as common *rpoB* mutations in a tuberculosis cohort in Bangladesh

**DOI:** 10.1099/mgen.0.001109

**Published:** 2023-09-26

**Authors:** Pauline Lempens, Armand Van Deun, Kya J. M. Aung, Mohammad A. Hossain, Mahboobeh Behruznia, Tom Decroo, Leen Rigouts, Bouke C. de Jong, Conor J. Meehan

**Affiliations:** ^1^​ Unit of Mycobacteriology, Department of Biomedical Sciences, Institute of Tropical Medicine, Antwerp, Belgium; ^2^​ Department of Biomedical Sciences, University of Antwerp, Antwerp, Belgium; ^3^​ Independent Consultant, Leuven, Belgium; ^4^​ Damien Foundation Bangladesh, Dhaka, Bangladesh; ^5^​ Department of Biosciences, Nottingham Trent University, Nottingham, UK; ^6^​ Unit of HIV and TB, Department of Clinical Sciences, Institute of Tropical Medicine, Antwerp, Belgium

**Keywords:** Mycobacterium, tuberculosis, Transmission, Drug resistance

## Abstract

The spread of multidrug-resistant tuberculosis (MDR-TB) is a growing problem in many countries worldwide. Resistance to one of the primary first-line drugs, rifampicin, is caused by mutations in the *Mycobacterium tuberculosis rpoB* gene. So-called borderline *rpoB* mutations confer low-level resistance, in contrast to more common *rpoB* mutations which confer high-level resistance. While some borderline mutations show lower fitness *in vitro* than common mutations, their *in vivo* fitness is currently unknown. We used a dataset of 394 whole genome sequenced MDR-TB isolates from Bangladesh, representing around 44 % of notified MDR-TB cases over 6 years, to look at differences in transmission clustering between isolates with borderline *rpoB* mutations and those with common *rpoB* mutations. We found a relatively low percentage of transmission clustering in the dataset (34.8 %) but no difference in clustering between different types of *rpoB* mutations. Compensatory mutations in *rpoA, rpoB,* and *rpoC* were associated with higher levels of transmission clustering as were lineages two, three, and four relative to lineage one. Young people as well as patients with high sputum smear positive TB were more likely to be in a transmission cluster. Our findings show that although borderline *rpoB* mutations have lower *in vitro* growth potential this does not translate into lower transmission potential or *in vivo* fitness. Proper detection of these mutations is crucial to ensure they do not go unnoticed and spread MDR-TB within communities.

## Data Summary

WGS reads are available in the European Nucleotide Archive (PRJEB39569). In addition, WGS reads, as well as pDST and clinical data, are included in the ReSeqTB data platform and are accessible on registration at https://platform.reseqtb.org/. Custom scripts for clustering are available at https://github.com/conmeehan/pathophy. An overview of the dataset is included in Supplementary Material 1 (Table S1) available in the online version of this article.

**Impact Statement**

Rifampicin is the primary first-line drug for treating tuberculosis, which is primarily caused by the bacterial pathogen *Mycobacterium tuberculosis *. Transmission of drug-resistant *M. tuberculosis* is not well understood, especially what effect the mutations that cause rifampicin resistance and the genetic background of the individual strain have on its transmissibility. In this study, we used a dataset of whole genome sequenced *M. tuberculosis* from Bangladesh to give insights into this area. We found that there was no difference in ability to transmit between strains with different rifampicin resistance-related mutations in *rpoB*. This means that even those mutations thought to affect the growth of strains *in vivo*, and that often are difficult to detect in the clinic, can still easily spread between patients. Also, we find that some lineages of *M. tuberculosis* transmit better than others, with lineage one specifically being associated with lower transmissibility. These findings indicate that to control rifampicin-resistant tuberculosis we need to ensure that we accurately detect all *rpoB* mutations and track their spread in the community.

## Introduction

Tuberculosis (TB) is an airborne disease caused by *Mycobacterium tuberculosis* (MTB). It is one of the main causes of death from an infectious disease worldwide [1]. Antimicrobial drug resistance hinders control of the TB pandemic. On average, about 3–4 % of newly diagnosed TB patients, and about 18–21 % of patients previously treated for TB have rifampicin-resistant TB (RR-TB) or multidrug-resistant TB (MDR-TB, resistant to rifampicin and isoniazid) [1]. Although rifampicin resistance testing coverage and RR/MDR-TB treatment success rates have increased in the last decade, the global RR/MDR-TB burden remains unchanged [1]. Continued efforts to improve our understanding and control of RR/MDR-TB are therefore required.

RR/MDR-TB was long believed to occur mainly through acquisition of resistance during first-line treatment [2]. Molecular studies have however shown that the vast majority of RR/MDR-TB is transmitted [2]. In recent years, whole genome sequencing (WGS) has added a new dimension to (RR/MDR-)TB epidemiological research, offering the opportunity to investigate the dynamics and determinants of (RR/MDR-)TB transmission in greater detail, thereby providing insights on its optimal control [3, 4].

By definition, RR/MDR-TB isolates have deleterious mutations in the RNA polymerase (*rpoB*) gene, an essential gene for MTB. Not surprisingly, Ser450Leu, the most common *rpoB* mutation in MTB worldwide is also the one causing the least fitness loss, often accompanied by compensatory mutations in *rpoA* or *rpoC*, which can fully restore fitness [5, 6]. Less fit *rpoB* mutants tend to show *in vitro* growth defects that may lead to false rifampicin susceptibility in liquid based phenotypic drug susceptibility testing (pDST), while *in vivo* causing similarly high mortality and poor treatment outcome as Ser450Leu [7–10]. These so-called borderline mutations may spread more extensively in settings where the population is weakened, such as by HIV, further facilitated by inaccurate diagnostics not recognizing these strains as rifampicin resistant [11, 12]. We tested whether borderline mutations are as transmissible as ‘common’ *rpoB* mutations (i.e. similar transmission fitness) in a setting with low HIV co-infection where modern and ancestral lineages co-circulate.

## Methods

### Study population

MDR-TB patients from the Damien Foundation MDR-TB project area in Bangladesh, which covers 13 of 64 districts of the country, diagnosed between 2005 and 2011, treated with a standardized short treatment regimen, and with WGS data of their baseline *M. tuberculosis* isolate available were included [13, 14]. Details on diagnosis of MDR-TB and treatment regimen are described elsewhere [13, 14]. Patients with an *rpoB* wild-type baseline isolate or a baseline isolate containing only (an) *rpoB* mutation(s) with unknown association with rifampicin resistance (not listed in the ‘WHO Catalogue of mutations in *Mycobacterium tuberculosis* complex and their association with drug resistance’ and located outside the *rpoB* rifampicin resistance-determining region [RRDR] [codons 426–452]), were excluded [15].

### Whole genome sequencing

Most isolates (367/394) were included in our earlier publications about the Bangladesh MDR-TB cohort, and their WGS was described there (35 in Lempens *et al*., 2018 and 332 in Lempens *et al*., 2020) [16, 17]. The remaining 27 isolates were sequenced according to the same procedure as described in [16], with WGS done at the Translational Genomics Research Institute through the ReSeqTB sequencing platform [16, 18]. Non-MTB reads as identified by centrifuge were removed and isolates with >10 % non-MTB reads were excluded [19]. Quality control of the MTB reads was done using the MTBseq pipeline and isolates with <90 % coverage of the reference genome or an average sequencing depth <30× were excluded [20]. Command line version 2.8.12 of TBProfiler was used for read trimming and mapping, and variant calling and annotation [21, 22]. The literature-based TBProfiler library database (https://github.com/jodyphelan/tbdb) was accessed on 15 July 2020. In case of heteroresistance, variants of any frequency were included in the analysis. Drug resistance-associated variants with a sequencing depth below the default threshold of 10× but greater than 1× were included as well.

### Transmission clustering approach

Transmission clusters were calculated using a 5-SNP cut-off (which encompasses 5–10 years of transmission) [23] and a so-called loose (single linkage) clustering approach, in which isolates within a cluster had a maximum difference of five SNPs with at least one other isolate in the cluster [23]. To ensure only MDR-TB transmission was observed, clusters were further divided based on *rpoB* mutations, using an in-house developed Python script [24]. This meant that each transmission cluster contained only isolates with the same *rpoB* mutation, representing likely transmission of MDR-TB. If an isolate had one or more *rpoB* mutations in addition to the *rpoB* mutation shared with other isolates in the cluster, it was kept in the cluster. Custom scripts for clustering are available at https://github.com/conmeehan/pathophy.

### *rpoB* mutation classification

Based on their nonsynonymous *rpoB* mutations as reported by TBProfiler, isolates were divided into three groups: common, low-confidence and borderline. The ‘common *rpoB* mutations’ group consisted of isolates with one or more mutation(s) classified as ‘associated with resistance’ in the ‘WHO Catalogue of mutations in *Mycobacterium tuberculosis* complex and their association with drug resistance’ [15]. The ‘low-confidence *rpoB* mutations’ group contained isolates with one or more mutation(s) classified in the WHO catalogue as ‘associated with resistance interim’ because insufficient evidence on their association with rifampicin resistance exists. Their association with resistance is therefore based on the expert rule that any mutation within the *rpoB* RRDR (codons 426–452) should be assumed to confer resistance [15]. Using the same rule, we also included isolates with RRDR mutations not listed in the catalogue in the ‘low-confidence *rpoB* mutations’ group. Isolates with a combination of ‘associated with resistance interim’ or ‘RRDR’ mutations and ‘associated with resistance’ mutations (except 450Leu) were also included in the ‘low-confidence *rpoB* mutations’ group. The third group, the ‘borderline *rpoB* mutations’ group, consisted of isolates with a borderline mutation, alone or in combination with a second mutation. Based on the WHO catalogue, the following seven mutations were considered as borderline: Leu430Pro, Asp435Tyr, His445Asn, His445Leu, His445Ser, Leu452Pro, and Ile491Phe [15].

### Compensatory mutations

Some mutations in *rpoA*, *rpoB,* and/or *rpoC* have been shown to compensate for the fitness loss associated with rifampicin resistance-related mutations in *rpoB* [25]. We looked for the link between such mutations and transmission clustering in our dataset. We used two different definitions of potential compensatory mutations: an inclusive definition, which encompasses any mutation in *rpoA* or *rpoC*, and a confirmed definition, which encompasses only those mutations in *rpoA/B/C* that have been confirmed as compensatory by Gygli *et al*. [26]. These mutations were determined from the called files output from MTBseq.

### Statistics

Statistical analyses were carried out in R version 4.0.3 [27]. Multivariable logistic regression was carried out to investigate the association between type of *rpoB* mutation and presence in an MDR-TB transmission cluster. Other variables included were gender, age, sputum smear microscopy, lineage, fluoroquinolone resistance, and presence of a compensatory mutation. The model was simplified until all remaining variables were variables of interest or significantly associated with clustering. Significance *p*-value was set at 0.05.

## Results

Between 2005 and 2011, 894 patients were notified as diagnosed with MDR-TB in the Damien Foundation MDR-TB project area. Of 894 patients, 581 started with a gatifloxacin-based standardized short treatment regimen, and 11/894 started with an ofloxacin-based regimen [13, 14]; the remaining patients were lost to follow up due to a variety of reasons such as discontinuing treatment with the Damien Foundation or further tests revealing NTM disease instead of TB. For 414 patients, whole genome sequencing data of a baseline isolate was available. One patient was counted twice, as two treatment episodes were included, after confirmation of reinfection with a strain that was not in a transmission cluster with the strain of the first infection. Nineteen isolates were excluded because their *rpoB* gene was wild-type and one isolate was excluded because the association between its *rpoB* mutation and rifampicin resistance was unknown and the mutation was located outside the RRDR. As a result, 394 isolates were included in the analysis (Fig. S1 and Table S1 in Supplementary Material 1).

Based on the 5-SNP cut-off with loose clustering approach, 38.3 % (151/394) of patients clustered into 43 transmission clusters, while 61.7 % (243/394) of patients had a unique genotype. Of the 43 clusters, six clusters were divided based on *rpoB* mutations (Table 1). This resulted in 40 transmission clusters of isolates with the same *rpoB* mutation. Of 394 patients, 34.8 % (137) were in one of these 40 transmission clusters, while 65.2 % (257) of patients did not cluster. Clusters had a median size of 2.5 patients (range 2 to 16) and the maximum distance between isolates ranged from zero to nine SNPs (Fig. S2 in Supplementary Material 1). In 2/40 clusters, one of the clustered isolates had a second *rpoB* mutation besides a Ser450Leu mutation shared within the cluster. In one case, this was a deletion within the RRDR (rpoB_c.1306_1308del) and in the other a Glu761Asp mutation (with unknown association with rifampicin resistance). In both cases, the cluster was not divided, but kept as such.

**Table 1.** Overview of 5-SNP cut-off clusters that were split based on *rpoB* mutation

Isolate ID	cluster no. 5-SNP cut-off	cluster no. 5-SNP cut-off + *rpoB* mutation	*rpoB* mutation
ITM2007-02228	10	8	Ser450Leu
ITM2007-03332	10	8	Ser450Leu
ITM2007-01784	10	8	Ser450Leu
ITM2008-03145	10	8	Ser450Leu
ITM2008-02377	10	none	Leu452Pro
ITM2008-04299	2	none	His445Asp
ITM2010-00380	2	none	Val170Phe, His445Tyr
ITM2009-02024	24	14	Ser450Leu
ITM2009-02592	24	14	Ser450Leu
ITM2010-02419	24	14	Ser450Leu
ITM2006-01934	24	none	His445Asp
ITM2007-01828	29	none	Gln432Lys
ITM2007-01340	29	none	Ile491Phe
ITM2009-03558	32	none	Ser450Gly
ITM2008-00665	32	none	Ser450Leu
ITM2009-03268	32	none	Ser450Trp
ITM2005-02880	8	29	Asp435Val
ITM2005-02896	8	29	Asp435Val
ITM2011-02382	8	7	Ser450Leu
ITM2005-02145	8	7	Ser450Leu
ITM2005-02168	8	7	Ser450Leu
ITM2008-04297	8	7	Ser450Leu
ITM2009-01725	8	7	Ser450Leu
ITM2009-03258	8	7	Ser450Leu
ITM2010-02405	8	7	Ser450Leu
ITM2011-00569	8	7	Ser450Leu
ITM2011-00971	8	7	Ser450Leu
ITM2011-00375	8	none	His445Asp
ITM2006-04070	8	none	His445Tyr
ITM2009-02014	8	none	Ile491Phe
ITM2008-00210	8	none	Ser450Trp

Of 394 isolates included, 322 had a common *rpoB* mutation, 13 had a low-confidence mutation, and 59 had a borderline mutation. Table S2 (Supplementary Material 1) outlines all combinations of *rpoB* mutations found and their classification as ‘common’, ‘low-confidence’ or ‘borderline’.

The majority of patients were male (69.5 %) and had a baseline isolate derived from a highly smear positive sputum sample (2+ or 3+) (88.6 %) (Table 2). Isolates having a borderline *rpoB* mutation and isolates having a low-confidence mutation were found in a cluster at a similar frequency (35.6 % [aOR 1.8; 95 % CI 0.90–3.5] and 38.5 % [aOR 2.3; 95 % CI 0.62–8.3], respectively) as isolates with a common *rpoB* mutation (34.5 %) (*p*-values 0.096 and 0.19 respectively; 51 % power to detect a difference). Of the 394 isolates, 51 were resistant to fluoroquinolones, of which 23 (45.1 %) were found in a cluster. Four clusters contained one fluoroquinolone-resistant isolate each. One cluster contained two fluoroquinolone-resistant isolates, with different *gyrAB* mutations. Seven clusters contained at least two isolates with the same *gyrAB* mutation, suggesting transmission of pre-XDR TB.

**Table 2.** Factors associated with transmission clustering. Isolates within a transmission cluster had a maximum difference of 5 SNPs with at least one other isolate in the cluster and had the same rpoB mutation.

					Logistic regression	
	Total n	In a cluster n (%)	Unique n	Unadjusted OR (95 % CI)	Adjusted OR (95 % CI)	*p* value
Total	394	137 (34.8)	257			
Gender					ns	
Male	274	83 (30.3)	191	1		
Female	120	54 (45.0)	66	1.9 (1.2–2.9)		
Age						
10–24	124	71 (57.3)	53	3.1 (2.0–5.1)	2.8 (1.7–4.6)	<0.001
25–44	187	56 (29.9)	131	1	1	
≥45	83	10 (12.0)	73	0.32 (0.15–0.64)	0.33 (0.15–0.68)	0.004
Sputum smear						
Negative/Scanty/1+	45	9 (20.0)	36	1	1	
2+/3+	349	128 (36.7)	221	2.3 (1.1–5.3)	2.3 (1.1–5.6)	0.042
Lineage						
Lineage 1	115	17 (14.8)	98	1	1	
Lineage 2	91	36 (39.6)	55	3.8 (2.0–7.5)	3.3 (1.6–6.9)	0.001
Lineage 3	69	24 (34.8)	45	3.1 (1.5–6.4)	2.5 (1.1–5.5)	0.023
Lineage 4	119	60 (50.4)	59	5.9 (3.2–11.3)	4.7 (2.4–9.6)	<0.001
Fluoroquinolone					ns	
Susceptible	343	114 (33.2)	229	1		
Resistant	51	23 (45.1)	28	1.7 (0.90–3.0)		
Type of rpoB mutation						
Common	322	111 (34.5)	211	1	1	
Low-confidence	13	5 (38.5)	8	1.2 (0.35–3.6)	2.3 (0.62–8.3)	0.19
Borderline	59	21 (35.6)	38	1.1 (0.58–1.9)	1.8 (0.90–3.5)	0.096
Any mutation in rpoA/C					ns	
Absent	290	98 (33.8)	192	1		
Present	104	39 (37.5)	65	1.2 (0.73–1.9)		
Confirmed* compensatory						
rpoA/B/C mutation						
Absent	351	118 (33.6 %)	233	1	1	
Present	43	19 (44.2 %)	24	1.6 (0.82–3.0)	2.4 (1.1–5.0)	0.025

*Confirmed by Gygli *et al*., 2021 [26]. NS: not significant.

**Fig. 1.**
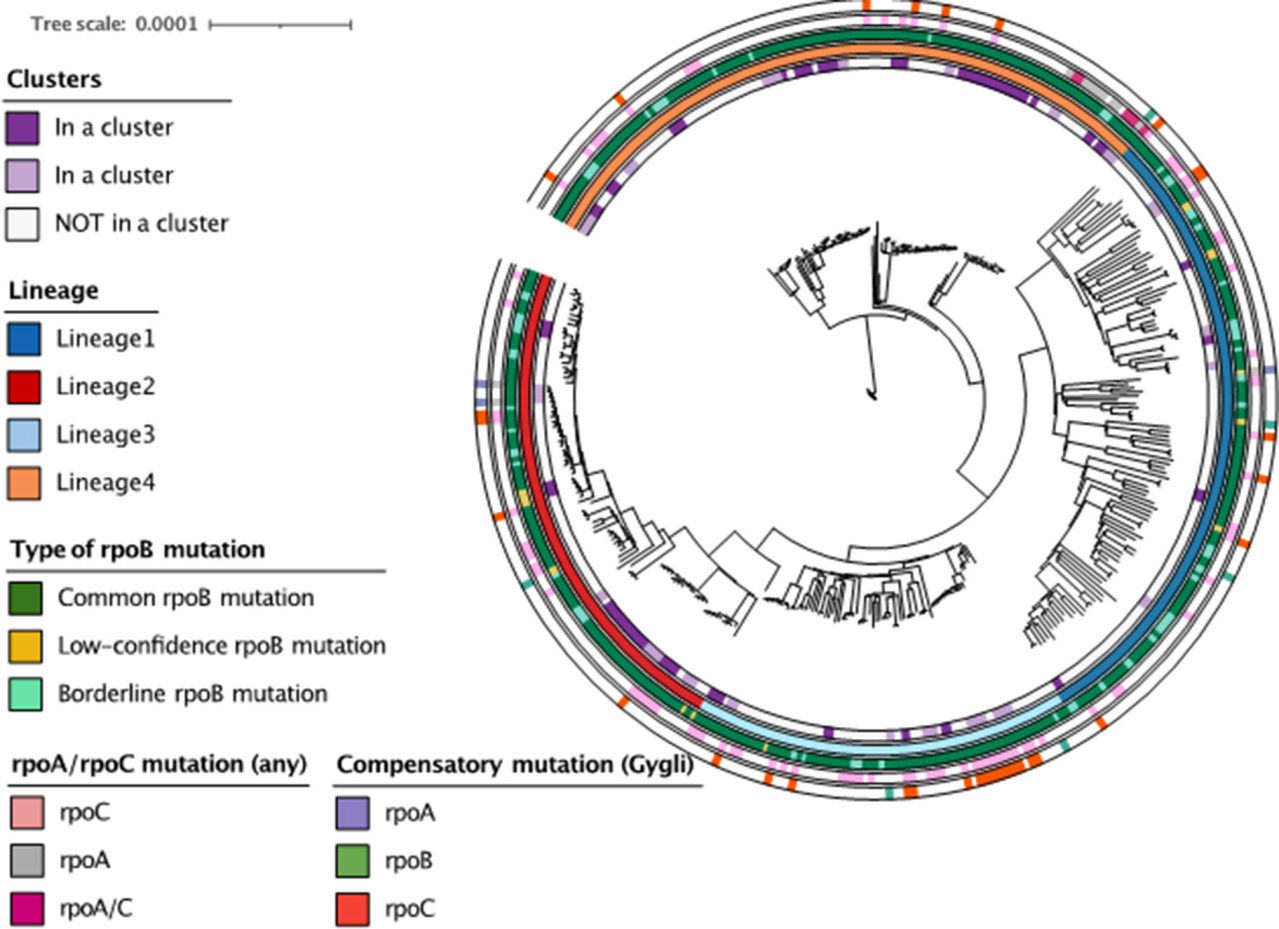
Maximum likelihood tree of the 394 isolates included in the study. Rings around the tree indicate the following (from inner to outer): 1) Clusters based on a 5-SNP cut-off combined with *rpoB* mutation, and are indicated in two shades of purple to allow for distinction between adjacent clusters; 2) Lineage of each samples; 3) Type of *rpoB* mutation; 4) Presence of any mutation in *rpoA* and/or *rpoC* ; 5) Presence of an *rpoA/B/C* mutation specifically found to be compensatory by Gygli *et al*. [26].

Isolates of modern *M. tuberculosis* lineage two (aOR 3.3; 95 % CI 1.6–6.9), lineage three (aOR 2.5; 95 % CI 1.1–5.5), and lineage four (aOR 4.7; 95 % CI 2.4–9.6) clustered significantly more often than isolates of ancestral lineage one (Table 2). In addition, people aged 10–24 years clustered significantly more often than people aged 25–44 years (aOR 2.8; 95 % CI 1.7–4.6), as did patients with highly sputum smear positive TB (2+ or 3+) compared to patients with a negative, scanty or 1+ sputum smear (aOR 2.3; 95 % CI 1.1–5.6). People aged ≥45 were significantly less likely to be in a cluster relative to people aged 25–44 years (aOR 0.33; 95 % CI 0.15–0.68). Strains with a confirmed compensatory mutation (based on [26, 26]) were significantly more likely to be in a transmission cluster (aOR 2.4; 95 % CI 1.1–5.0). Type of *rpoB* mutation, fluoroquinolone resistance, and presence of any mutations in *rpoA* or *rpoC* were not associated with being in a transmission cluster. Fig. 1 shows a maximum likelihood tree of the isolates included in the study, with the clusters, as well as lineage and type of *rpoB* and compensatory mutation depicted in circles around the tree.

## Discussion

In our 2005–2011 MDR-TB cohort in the Bangladesh Damien Foundation MDR-TB project, 34.8 % of MDR-TB resulted from transmission, with a median cluster size of 2.5 patients (range 2 to 16). Isolates with a borderline *rpoB* mutation and isolates with a low-confidence *rpoB* mutation clustered at a similar frequency (35.6 and 38.5 %, respectively) as isolates having a common *rpoB* mutation (34.5 %). Modern *M. tuberculosis* lineage (lineage 2, 3, or 4), young age, high sputum smear positivity, and compensatory mutations were associated with clustering.

Our results indicate that despite the *in vitro* fitness cost associated with borderline *rpoB* mutations, strains with such mutations seem to transmit equally well as strains with common *rpoB* mutations. In a drug-resistant TB cohort in Southern Brazil, 14/22 isolates (63.6 %) with a borderline *rpoB* mutation were found in a cluster, compared to an overall clustering proportion of MDR-TB isolates of 73.4 % [28]. Why these mutations are still seen less frequently than common *rpoB* mutations still needs to be explored, but may be due to other *in vivo* replicative fitness costs within the host.

We also find that compensatory mutations correlate positively with transmission clusters, irrespective of the underlying *rpoB* mutation. This suggests that all *rpoB* mutations benefit from compensatory mutations in *rpoA/B/C* to reduce fitness loss. However, labelling any mutation in *rpoA* or *rpoC* as compensatory did not correlate with transmission. This highlights that more understanding is needed into which specific mutations can compensate for fitness loss associated with antibiotic resistance as this is likely underestimated at present.

In addition to compensatory mutations, isolates with low-confidence or borderline *rpoB* mutations may benefit from obtaining a second *rpoB* mutation, with net advantage of increased rifampicin resistance compared to combined fitness loss [29]. When a low-confidence or borderline *rpoB* mutation and a second *rpoB* mutation are acquired in the same genome one after another, we hypothesize that the low-confidence or borderline *rpoB* mutation was acquired prior to the second *rpoB* mutation. As a consequence, the effect on transmission could not have been present without the first sequential variant, characterised by the highest proportion mutant (unless both become fixed). Therefore, we included isolates with a combination of ‘associated with resistance interim’ or ‘RRDR’ mutations and ‘associated with resistance’ mutations (except 450Leu) in the low-confidence group. Likewise, isolates with a combination of a borderline mutations and a second *rpoB* mutation were included in the borderline group.

Besides early and accurate detection of rifampicin resistance, effective treatment is crucial to stop RR/MDR-TB transmission. In the Bangladesh Damien Foundation MDR-TB project area, a standardized, highly effective treatment regimen has been in place for several decades now. In settings such as this, rifampicin resistance-associated variants that are more difficult to detect, may become the future TB endemic while variants that are detected and treated, are reduced. While the introduction of GeneXpert enables better detection of borderline *rpoB* mutations located within the RRDR, it misses mutations outside the RRDR (e.g. Ile491Phe).

The availability of effective RR/MDR-TB treatment in the Bangladesh Damien Foundation MDR-TB project area may explain the relatively low percentage of isolates that clustered. In a nationwide drug-resistant TB (DR-TB) surveillance study conducted in Bangladesh between 2011 and 2017 and 38.4 % of MDR-TB isolates were found in a cluster [30]. Our results are in line with these findings, despite the use of different transmission cluster estimation techniques (spoligotyping and 24-locus Mycobacterial Interspersed Repetitive Unit Variable Number Tandem Repeat vs WGS). Higher clustering percentages of MDR-TB isolates were reported in similar studies in Southern Brazil (73.4 %), Tunisia (65.2 %), and Saudi Arabia (67.6 %), and of XDR-TB isolates in South Africa (85.5 %) [24, 28, 31, 32]. In contrast, clustering percentages similar to the one found in our study were reported in Shenzhen (25.2 %) and Shanghai (31.8 %), in China [33, 34].

Lineages in the *Mycobacterium tuberculosis* complex are increasingly recognized to have co-evolved with humans, with recent lineages adapted to higher population densities where rapid spread is advantageous [35–37]. The modern lineages two, three, and four led to more secondary cases than the ancient lineages one and six [38, 39]. Our results suggest that known differences in transmissibility between *M. tuberculosis* lineages also apply to MDR-TB strains. In the DR-TB surveillance study in Bangladesh mentioned above, a significantly higher clustering rate was found in modern lineage isolates (49.3 %) compared to ancient lineage isolates (9.6 %) [30]. In an MDR-TB cohort in Saudi Arabia consisting of isolates belonging to lineage 1–4, no difference in clustering proportion was found between lineages [32]. However, since immigration rates in this setting were high (43.7 % of patients were non-Saudi), transmission differences between lineages may not have been visible due to differences in lineage distribution between the highly diverse populations in the country. An alternative (partial) explanation for our findings could be the fact that lineage one, and to a lesser extent lineage two have a higher molecular clock rate than lineage four [40, 41]. Using the same SNP cut-off for all lineages could therefore have led to an underestimation of transmission events of lineage one isolates compared to lineage four isolates [40].

The predominance of young people (age 10–24 years) in clusters is probably explained by recent industrialization in the Damien Foundation project area. Factories started to be built in large numbers around the country’s capital Dhaka not so long before the study period, and by now extend into the Southern part of the project area. From the project’s main MDR-TB detection districts in Greater Mymensingh, young people moved to these factory areas for work. Overcrowded living conditions and lack of employment protection when reporting ill, leading to self-medication with over-the-counter antibiotics, promote not only transmission but also creation of drug-resistant tuberculosis [42].

Our study has a few limitations. Firstly, not all RR-/MDR-TB patients in the Damien Foundation project area were diagnosed, started treatment, and had whole genome sequencing data available (Fig. S1 in Supplementary Material 1). A caveat with transmission studies is that incomplete sampling fractions, as well as limited duration, tend to overestimate ‘unique’ isolates and thus underestimate TB due to recent transmission, as does migration into an area. While transmission to and from the area dense with garment manufacturers may not have been fully captured, the population based nature and 6 year study duration support that a low clustering rate may indeed be due to adequate detection and treatment of MDR-TB. Secondly, besides the explanatory factors included in this study, other factors known to be associated with transmission of TB such as co-morbidities and host malnutrition were not assessed [43]. HIV-infection prevalence in the study setting is very low (<1 %). Transmission of borderline *rpoB* mutants was likely underestimated since rifampicin resistance in these isolates more often remained undetected in the pre-GeneXpert study period and thus were not subjected to WGS. In addition, whole genome sequencing was done from cultured isolates, which may have led to culture bias affecting mutations with a relatively high fitness cost. Our study also revealed many small transmission clusters, which precluded more in-depth analysis of transmission dynamics such as timing emergence of resistance and compensatory mutations. Further study into larger clusters of non-lineage four isolates will help us understand these dynamics better. Finally, estimation of transmission timespans encompassed by SNP cut-offs of many lineages are not as well defined as for lineage four [23], meaning small over or under estimations can occur. Extensive transmission studies in lineages one and three will help refine these estimates in the future.

Transmission fitness is a complex phenotype, without straight correlation between replicative (e.g. *in vitro*) fitness and transmission (e.g. *in vivo*) fitness [29, 44]. Future studies that include data on the fitness cost of individual *rpoB* mutations and combinations of mutations could shed more light on the effect of fitness cost on transmission.

In conclusion, isolates with a borderline *rpoB* mutation and isolates with a low-confidence *rpoB* mutation transmitted equally well as isolates having a common *rpoB* mutation in our cohort of MDR-TB patients from Bangladesh. In addition, modern *M. tuberculosis* lineage two, three, and four were associated with transmission. Our findings stress the importance of accurate detection of resistant variants in order to maximally limit their spread.

## Supplementary Data

Supplementary material 1
