# Supplementary material for: Borderline rpoB mutations transmit at the same rate as common rpoB mutations in a tuberculosis cohort in Bangladesh
Source: Microb Genom. 2023 Sep 26;9(9):001109. doi: 10.1099/mgen.0.001109 (PMC10569737; doi:10.1099/mgen.0.001109)

Supplementary Table 1. Study dataset. This is also available as an excel sheet via Figshare: https://doi.org/10.6084/m9.figshare.23902311

isolateID	ENAaccession	gender	age	dateSputumCollection	sputumSmear	clusters5SNP	clusters5SNP_RMPpattern	clusters5SNP_RMPpattern_YesNo	lineage	sublineage	sublineage_1stLevel	rpoBmutation(s)	rpoB_Classification	rpoAC	rpoACYesNo	Gygli	GygliYesNo	fluoroquinolonesRS
ITM2011-01502	ERR4553880	F	21	16/05/2011	3+	c1	c1	yes	lineage4	lineage4.8	lineage4.8	rpoB_p.Ser450Trp	common	no	no	no	no	R
ITM2006-01934	ERR4553498	M	29	20/04/2006	2+	c24	removed	no	lineage3	lineage3.1	lineage3.1	rpoB_p.His445Asp	common	no	no	no	no	S
ITM2007-01338	ERR4553535	M	50	23/01/2007	1+	c26	c30	yes	lineage4	lineage4.3.4.2	lineage4.3	rpoB_p.Asp435Val	common	no	no	no	no	S
ITM2011-02587	ERR4553889	F	21	26/10/2011	3+	c3	c2	yes	lineage2	lineage2.2	lineage2.2	rpoB_p.Ser450Leu	common	no	no	no	no	R
ITM2008-04057	ERR4553665	M	22	02/09/2008	3+	c5	c4	yes	lineage4	lineage4	lineage4	rpoB_p.Ser450Leu	common	no	no	no	no	R
ITM2008-03358	ERR4553659	M	35	21/06/2008	3+	c6	c5	yes	lineage2	lineage2.2	lineage2.2	rpoB_p.Ser450Leu	common	no	no	no	no	R
ITM2005-02085	ERR4553436	F	22	01/06/2005	2+	c7	c6	yes	lineage4	lineage4.3.4.2	lineage4.3	rpoB_p.Ser450Leu	common	no	no	no	no	R
ITM2011-02404	ERR4553887	M	25	21/09/2011	3+	none	none	no	lineage4	lineage4	lineage4	rpoB_p.Ser450Leu	common	rpoC	yes	rpoC	yes	R
ITM2006-01165	ERR4553494	F	31	19/02/2006	3+	none	none	no	lineage3	lineage3	lineage3	rpoB_p.Asp435Val	common	rpoC	yes	no	no	R
ITM2006-04061	ERR4553523	M	20	13/04/2006	2+	none	none	no	lineage1	lineage1.2.2.2	lineage1.2	rpoB_p.Ser450Leu	common	no	no	no	no	S
ITM2008-02050	ERR4553636	M	35	16/03/2008	2+	none	none	no	lineage2	lineage2.2.1	lineage2.2	rpoB_p.Asp435Tyr	borderline	no	no	no	no	S
ITM2005-01552	ERR4553427	M	24	10/04/2005	3+	none	none	no	lineage4	lineage4.3.4.2	lineage4.3	rpoB_p.Ser450Leu	common	rpoC	yes	rpoC	yes	R
ITM2005-01580	ERR4553429	M	29	28/03/2005	2+	none	none	no	lineage1	lineage1.2.2	lineage1.2	rpoB_p.Asp435Tyr	borderline	no	no	no	no	R
ITM2005-02144	ERR4553440	M	45	16/06/2005	2+	none	none	no	lineage2	lineage2.2.1	lineage2.2	rpoB_p.Leu430Pro	borderline	no	no	no	no	S
ITM2005-02151	ERR4553442	F	35	04/07/2005	2+	none	none	no	lineage4	lineage4.3.4.2	lineage4.3	rpoB_p.Leu452Pro	borderline	no	no	no	no	S
ITM2005-02879	ERR4553459	F	25	25/10/2005	2+	none	none	no	lineage1	lineage1.2.2	lineage1.2	rpoB_p.Val170Phe	common	no	no	no	no	S
ITM2006-00626	ERR4553481	M	40	06/02/2006	2+	none	none	no	lineage1	lineage1.1.3	lineage1.1	rpoB_p.His445Asp	common	no	no	no	no	R
ITM2006-04048	ERR4553520	F	37	21/09/2006	1+	none	none	no	lineage2	lineage2.2.1	lineage2.2	rpoB_p.His445Arg	common	no	no	no	no	S
ITM2007-01747	ERR4553548	M	22	07/02/2007	1+	none	none	no	lineage1	lineage1.1.3.1	lineage1.1	rpoB_p.Leu452Pro	borderline	no	no	no	no	S
ITM2007-02423	ERR4553570	F	27	20/05/2007	3+	none	none	no	lineage1	lineage1.1.3.1	lineage1.1	rpoB_p.Ser450Leu	common	no	no	no	no	S
ITM2008-02742	ERR4553644	M	70	09/04/2008	3+	none	none	no	lineage1	lineage1.1.3.3	lineage1.1	rpoB_p.Ser450Leu	common	no	no	no	no	S
ITM2008-02753	ERR4553645	F	41	04/05/2008	negative	none	none	no	lineage1	lineage1.1.3.1	lineage1.1	rpoB_p.Ser450Leu	common	rpoC	yes	no	no	S
ITM2008-04298	ERR4553674	M	26	27/10/2008	3+	none	none	no	lineage1	lineage1.2.2.2	lineage1.2	rpoB_p.Ser450Leu	common	no	no	no	no	S
ITM2012-00034	ERR4553890	M	21	31/10/2011	3+	none	none	no	lineage2	lineage2.2.1	lineage2.2	rpoB_p.Ser450Leu	common	rpoC	yes	no	no	R
ITM2012-00036	ERR4553891	M	23	02/11/2011	1+	none	none	no	lineage2	lineage2.2.1	lineage2.2	rpoB_p.Ser450Leu	common	no	no	no	no	R
ITM2012-00341	ERR4553892	F	10	14/12/2011	3+	none	none	no	lineage1	lineage1.1.3.1	lineage1.1	rpoB_p.Leu430Pro	borderline	no	no	no	no	S
ITM2010-00271	ERR4553759	M	27	04/11/2009	2+	c1	c1	yes	lineage4	lineage4.8	lineage4.8	rpoB_p.Ser450Trp	common	no	no	no	no	R
ITM2010-00389	ERR4553765	M	31	20/12/2009	3+	c1	c1	yes	lineage4	lineage4.8	lineage4.8	rpoB_p.Ser450Trp	common	no	no	no	no	S
ITM2011-01203	ERR4553866	F	16	09/03/2011	2+	c1	c1	yes	lineage4	lineage4.8	lineage4.8	rpoB_p.Ser450Trp	common	no	no	no	no	R
ITM2007-02228	ERR4553567	M	23	22/05/2007	2+	c10	c8	yes	lineage2	lineage2.2.1	lineage2.2	rpoB_p.Ser450Leu	common	no	no	no	no	S
ITM2007-03332	ERR4553585	M	15	01/08/2007	2+	c10	c8	yes	lineage2	lineage2.2.1	lineage2.2	rpoB_p.Ser450Leu	common	no	no	no	no	R
ITM2007-01784	ERR4553551	M	21	26/02/2007	2+	c10	c8	yes	lineage2	lineage2.2.1	lineage2.2	rpoB_p.Ser450Leu	common	rpoA	yes	rpoA	yes	S
ITM2008-02377	ERR4553639	M	30	03/04/2008	3+	c10	removed	no	lineage2	lineage2.2.1	lineage2.2	rpoB_p.Leu452Pro	borderline	no	no	no	no	S
ITM2008-03145	ERR4553654	M	40	22/06/2008	3+	c10	c8	yes	lineage2	lineage2.2.1	lineage2.2	rpoB_p.Ser450Leu	common	rpoA	yes	rpoA	yes	S
ITM2008-03135	ERR4553653	F	18	07/04/2008	3+	c11	c9	yes	lineage3	lineage3	lineage3	rpoB_p.Ser450Leu	common	no	no	no	no	R
ITM2006-01035	ERR4553492	M	42	07/02/2006	2+	c11	c9	yes	lineage3	lineage3	lineage3	rpoB_p.Ser450Leu	common	rpoC	yes	rpoC	yes	S
ITM2006-01944	ERR4553502	M	44	14/05/2006	2+	c11	c9	yes	lineage3	lineage3	lineage3	rpoB_p.Ser450Leu	common	rpoC	yes	rpoC	yes	S
ITM2007-02217	ERR4553565	F	21	08/05/2007	3+	c11	c9	yes	lineage3	lineage3	lineage3	rpoB_p.Ser450Leu	common	rpoC	yes	rpoC	yes	S
ITM2010-00227	ERR4553755	M	32	01/10/2009	2+	c11	c9	yes	lineage3	lineage3	lineage3	rpoB_p.Ser450Leu	common	rpoC	yes	rpoC	yes	S
ITM2011-00062	ERR4553829	M	30	22/09/2010	2+	c11	c9	yes	lineage3	lineage3	lineage3	rpoB_p.Ser450Leu	common	rpoC	yes	rpoC	yes	S
ITM2011-00339	ERR4553840	M	40	18/10/2010	3+	c11	c9	yes	lineage3	lineage3	lineage3	rpoB_p.Ser450Leu	common	rpoC	yes	rpoC	yes	R
ITM2011-01237	ERR4553869	M	28	28/02/2011	1+	c11	c9	yes	lineage3	lineage3	lineage3	rpoB_p.Ser450Leu	common	rpoC	yes	rpoC	yes	S
ITM2008-04048	ERR4553663	M	23	18/09/2008	3+	c12	c10	yes	lineage3	lineage3.1	lineage3.1	rpoB_p.Ser450Leu	common	no	no	no	no	S
ITM2008-03704	ERR4553661	F	21	17/08/2008	3+	c12	c10	yes	lineage3	lineage3.1	lineage3.1	rpoB_p.Ser450Leu	common	no	no	no	no	S
ITM2009-00798	ERR4553689	M	25	12/11/2008	3+	c13	c11	yes	lineage3	lineage3	lineage3	rpoB_p.Ser450Leu	common	no	no	no	no	R
ITM2007-01731	ERR4553545	M	25	18/01/2007	3+	c13	c11	yes	lineage3	lineage3	lineage3	rpoB_p.Ser450Leu	common	no	no	rpoB	yes	R
ITM2005-01588	ERR4553430	M	37	28/03/2005	3+	c14	c39	yes	lineage1	lineage1.2.2	lineage1.2	rpoB_p.Met434Thr,rpoB_p.Asp435Gly	inferred	no	no	no	no	S
ITM2007-01868	ERR4553555	M	26	05/04/2007	2+	c14	c39	yes	lineage1	lineage1.2.2.2	lineage1.2	rpoB_p.Met434Thr,rpoB_p.Asp435Gly	inferred	no	no	no	no	S
ITM2005-02137	ERR4553439	M	24	05/06/2005	2+	c15	c24	yes	lineage2	lineage2.2	lineage2.2	rpoB_p.His445Tyr	common	rpoC	yes	no	no	S
ITM2006-02538	ERR4553512	M	18	29/06/2006	2+	c15	c24	yes	lineage2	lineage2.2	lineage2.2	rpoB_p.His445Tyr	common	rpoC	yes	no	no	S
ITM2010-01641	ERR4553780	M	24	09/03/2010	2+	c15	c24	yes	lineage2	lineage2.2	lineage2.2	rpoB_p.His445Tyr	common	rpoC	yes	no	no	S
ITM2011-01359	ERR4553875	M	16	11/04/2011	3+	c15	c24	yes	lineage2	lineage2.2	lineage2.2	rpoB_p.His445Tyr	common	rpoC	yes	no	no	S
ITM2005-02427	ERR4553448	M	24	01/08/2005	3+	c16	c31	yes	lineage4	lineage4	lineage4	rpoB_p.Ser428Thr,rpoB_p.His445Leu	borderline	no	no	no	no	S
ITM2006-03454	ERR4553515	M	32	10/07/2006	3+	c16	c31	yes	lineage4	lineage4	lineage4	rpoB_p.Ser428Thr,rpoB_p.His445Leu	borderline	no	no	no	no	S
ITM2008-04276	ERR4553670	F	36	21/10/2008	3+	c16	c31	yes	lineage4	lineage4	lineage4	rpoB_p.Ser428Thr,rpoB_p.His445Leu	borderline	no	no	no	no	S
ITM2005-02678	ERR4553451	M	35	11/09/2005	2+	c17	c32	yes	lineage2	lineage2.2.1	lineage2.2	rpoB_p.Thr427Pro,rpoB_p.His445Tyr	inferred	no	no	no	no	S
ITM2007-01883	ERR4553557	M	20	28/04/2007	3+	c17	c32	yes	lineage2	lineage2.2.1	lineage2.2	rpoB_p.Thr427Pro,rpoB_p.His445Tyr	inferred	no	no	no	no	S
ITM2010-02096	ERR4553793	M	41	23/05/2010	3+	c17	c32	yes	lineage2	lineage2.2.1	lineage2.2	rpoB_p.Thr427Pro,rpoB_p.His445Tyr	inferred	no	no	no	no	S
ITM2005-02826	ERR4553456	M	32	04/10/2005	3+	c18	c37	yes	lineage4	lineage4.9	lineage4.9	rpoB_p.His445Asn	borderline	no	no	no	no	S
ITM2008-01710	ERR4553624	M	36	29/01/2008	3+	c18	c37	yes	lineage4	lineage4.9	lineage4.9	rpoB_p.His445Asn	borderline	no	no	no	no	S
ITM2010-01936	ERR4553791	F	15	23/05/2010	3+	c18	c37	yes	lineage4	lineage4.9	lineage4.9	rpoB_p.His445Asn	borderline	no	no	no	no	S
ITM2005-02873	ERR4553457	F	20	26/10/2005	scanty	c19	c34	yes	lineage4	lineage4.1.1.1	lineage4.1	rpoB_p.Asp435Tyr,rpoB_p.Ser431Asn	borderline	rpoA	yes	no	no	S
ITM2011-00076	ERR4553831	F	26	28/09/2010	3+	c19	c34	yes	lineage4	lineage4.1.1.1	lineage4.1	rpoB_p.Asp435Tyr	borderline	rpoA	yes	no	no	S
ITM2010-00380	ERR4553763	M	38	11/11/2009	2+	c2	removed	no	lineage2	lineage2.2.1	lineage2.2	rpoB_p.Val170Phe,rpoB_p.His445Tyr	common	no	no	no	no	R
ITM2008-04299	ERR4553675	M	54	14/10/2008	3+	c2	removed	no	lineage2	lineage2.2.1	lineage2.2	rpoB_p.His445Asp	common	no	no	no	no	S
ITM2005-02939	ERR4553466	F	30	11/10/2005	3+	c20	c12	yes	lineage3	lineage3	lineage3	rpoB_p.Ser450Leu	common	rpoC	yes	rpoC	yes	S
ITM2010-02612	ERR4553817	F	30	10/08/2010	1+	c20	c12	yes	lineage3	lineage3	lineage3	rpoB_p.Ser450Leu	common	rpoC	yes	rpoC	yes	S
ITM2005-02951	ERR4553467	M	27	09/11/2005	2+	c21	c13	yes	lineage4	lineage4.1.1.1	lineage4.1	rpoB_p.Ser450Leu	common	rpoArpoC	yes	no	no	S
ITM2010-00888	ERR4553769	F	35	18/01/2010	3+	c21	c13	yes	lineage4	lineage4.1.1.1	lineage4.1	rpoB_p.Ser450Leu	common	rpoArpoC	yes	no	no	S
ITM2006-00662	ERR4553483	M	49	01/01/2006	3+	c22	c35	yes	lineage2	lineage2.2.1	lineage2.2	rpoB_p.Asp435Tyr,rpoB_p.Met434Arg	borderline	no	no	no	no	S
ITM2008-01320	ERR4553620	F	36	19/12/2007	3+	c22	c35	yes	lineage2	lineage2.2.1	lineage2.2	rpoB_p.Asp435Tyr	borderline	no	no	no	no	S
ITM2009-00815	ERR4553692	M	51	05/01/2009	2+	c22	c35	yes	lineage2	lineage2.2.1	lineage2.2	rpoB_p.Asp435Tyr	borderline	no	no	no	no	S
ITM2006-01009	ERR4553490	M	36	09/02/2006	2+	c23	c26	yes	lineage1	lineage1.1.3.1	lineage1.1	rpoB_p.Leu430Pro	borderline	no	no	no	no	S
ITM2009-03757	ERR4553749	F	37	13/09/2009	3+	c23	c26	yes	lineage1	lineage1.1.3	lineage1.1	rpoB_p.Leu430Pro	borderline	no	no	no	no	S
ITM2009-02024	ERR4553715	M	24	19/04/2009	2+	c24	c14	yes	lineage3	lineage3.1	lineage3.1	rpoB_p.Ser450Leu	common	rpoC	yes	no	no	S
ITM2009-02592	ERR4553733	M	19	19/07/2009	1+	c24	c14	yes	lineage3	lineage3.1	lineage3.1	rpoB_p.Ser450Leu	common	rpoC	yes	no	no	S
ITM2010-02419	ERR4553811	F	22	22/07/2010	2+	c24	c14	yes	lineage3	lineage3.1	lineage3.1	rpoB_p.Ser450Leu	common	rpoC	yes	no	no	S
ITM2006-03444	ERR4553513	M	25	24/07/2006	1+	c25	c15	yes	lineage4	lineage4.1.1.1	lineage4.1	rpoB_p.Ser450Leu	common	rpoA	yes	rpoB	yes	S
ITM2008-01314	ERR4553618	M	25	21/01/2008	3+	c25	c15	yes	lineage4	lineage4.1.1.1	lineage4.1	rpoB_p.Ser450Leu	common	rpoArpoC	yes	no	no	S
ITM2011-00540	ERR4553848	F	16	19/12/2010	3+	c25	c15	yes	lineage4	lineage4.1.1.1	lineage4.1	rpoB_p.Ser450Leu	common	rpoArpoC	yes	rpoC	yes	S
ITM2006-04059	ERR4553522	M	20	09/10/2006	2+	c26	c30	yes	lineage4	lineage4.3.4.2	lineage4.3	rpoB_p.Asp435Val	common	no	no	no	no	S
ITM2007-00054	ERR4553525	M	27	05/11/2006	2+	c27	c16	yes	lineage1	lineage1.2.2.2	lineage1.2	rpoB_p.Ser450Leu	common	no	no	no	no	S

ITM2007-00073	ERR4553527	M	60	08/11/2006	3+	c27	c16	yes	lineage1	lineage1.2.2.2	lineage1.2	rpoB_p.Ser450Leu	common	no	no	no	no	S
ITM2007-00057	ERR4553526	F	18	30/10/2006	3+	c28	c40	yes	lineage2	lineage2.2.1	lineage2.2	rpoB_c.1306_1308del,rpoB_p.Ser450Leu	common	no	no	no	no	S
ITM2007-01355	ERR4553538	M	23	07/02/2007	3+	c28	c40	yes	lineage2	lineage2.2.1	lineage2.2	rpoB_p.Ser450Leu	common	no	no	no	no	S
ITM2007-01340	ERR4553536	M	46	27/01/2007	1+	c29	removed	no	lineage2	lineage2.2.1	lineage2.2	rpoB_p.Ile491Phe	borderline	no	no	no	no	S
ITM2007-01828	ERR4553552	M	37	25/01/2007	1+	c29	removed	no	lineage2	lineage2.2.1	lineage2.2	rpoB_p.Gln432Lys	common	no	no	no	no	S
ITM2010-01140	ERR4553774	F	21	23/02/2010	3+	c3	c2	yes	lineage2	lineage2.2	lineage2.2	rpoB_p.Ser450Leu	common	no	no	no	no	R
ITM2007-01337	ERR4553534	M	18	23/01/2007	2+	c3	c2	yes	lineage2	lineage2.2.2	lineage2.2	rpoB_p.Ser450Leu	common	no	no	no	no	S
ITM2007-03189	ERR4553578	M	16	30/07/2007	3+	c3	c2	yes	lineage2	lineage2.2.2	lineage2.2	rpoB_p.Ser450Leu	common	no	no	no	no	S
ITM2007-03258	ERR4553579	F	16	24/07/2007	3+	c3	c2	yes	lineage2	lineage2.2.2	lineage2.2	rpoB_p.Ser450Leu	common	no	no	no	no	S
ITM2008-00217	ERR4553603	F	15	17/11/2007	2+	c3	c2	yes	lineage2	lineage2.2	lineage2.2	rpoB_p.Ser450Leu	common	no	no	no	no	S
ITM2008-01779	ERR4553630	F	40	28/01/2008	1+	c3	c2	yes	lineage2	lineage2.2	lineage2.2	rpoB_p.Ser450Leu	common	no	no	no	no	S
ITM2008-04042	ERR4553662	F	16	25/08/2008	3+	c3	c2	yes	lineage2	lineage2.2	lineage2.2	rpoB_p.Ser450Leu	common	no	no	no	no	S
ITM2009-02334	ERR4553725	F	40	13/06/2009	scanty	c3	c2	yes	lineage2	lineage2.2	lineage2.2	rpoB_p.Ser450Leu	common	no	no	no	no	S
ITM2010-02609	ERR4553816	F	28	03/08/2010	2+	c3	c2	yes	lineage2	lineage2.2	lineage2.2	rpoB_p.Ser450Leu	common	no	no	no	no	S
ITM2010-02800	ERR4553825	F	40	20/09/2010	2+	c3	c2	yes	lineage2	lineage2.2	lineage2.2	rpoB_p.Ser450Leu	common	no	no	no	no	S
ITM2007-01395	ERR4553541	M	21	28/12/2006	3+	c30	c36	yes	lineage1	lineage1.1.3.1	lineage1.1	rpoB_p.Asp435Tyr	borderline	no	no	no	no	S
ITM2008-00255	ERR4553607	M	41	31/10/2007	3+	c30	c36	yes	lineage1	lineage1.1.3.1	lineage1.1	rpoB_p.Asp435Tyr	borderline	no	no	no	no	S
ITM2007-02197	ERR4553561	M	20	10/05/2007	2+	c31	c17	yes	lineage3	lineage3	lineage3	rpoB_p.Ser450Leu	common	rpoC	yes	no	no	S
ITM2010-00887	ERR4553768	F	23	14/01/2010	3+	c31	c17	yes	lineage3	lineage3	lineage3	rpoB_p.Ser450Leu	common	rpoC	yes	no	no	S
ITM2011-00543	ERR4553849	F	35	13/12/2010	3+	c31	c17	yes	lineage3	lineage3	lineage3	rpoB_p.Ser450Leu	common	rpoC	yes	no	no	S
ITM2008-00665	ERR4553610	F	16	26/12/2007	1+	c32	removed	no	lineage1	lineage1.1.2	lineage1.1	rpoB_p.Ser450Leu	common	no	no	no	no	S
ITM2009-03268	ERR4553744	F	30	16/08/2009	1+	c32	removed	no	lineage1	lineage1.1.2	lineage1.1	rpoB_p.Ser450Trp	common	no	no	no	no	S
ITM2009-03558	ERR4553746	F	36	22/09/2009	3+	c32	removed	no	lineage1	lineage1.1.2	lineage1.1	rpoB_p.Ser450Gly	inferred	no	no	rpoB	yes	S
ITM2008-02054	ERR4553638	M	61	28/02/2008	3+	c33	removed	no	lineage2	lineage2.2.1	lineage2.2	rpoB_p.His445Tyr	common	no	no	no	no	S
ITM2008-03082	ERR4553647	M	18	04/06/2008	2+	c34	c18	yes	lineage3	lineage3	lineage3	rpoB_p.Ser450Leu	common	no	no	no	no	S
ITM2009-01727	ERR4553705	M	25	20/04/2009	2+	c34	c18	yes	lineage3	lineage3	lineage3	rpoB_p.Ser450Leu	common	no	no	no	no	S
ITM2008-04099	ERR4553669	F	45	03/09/2008	3+	c35	c19	yes	lineage4	lineage4.3.4.2	lineage4.3	rpoB_p.Ser450Leu	common	no	no	no	no	S
ITM2009-02346	ERR4553728	M	18	13/06/2009	3+	c35	c19	yes	lineage4	lineage4.3.4.2	lineage4.3	rpoB_p.Ser450Leu	common	no	no	no	no	S
ITM2011-00390	ERR4553844	M	24	04/11/2010	3+	c35	c19	yes	lineage4	lineage4.3.4.2	lineage4.3	rpoB_p.Ser450Leu	common	rpoC	yes	no	no	S
ITM2008-04336	ERR4553679	M	22	01/09/2008	3+	c36	c38	yes	lineage1	lineage1.2.2	lineage1.2	rpoB_p.His445Asn	borderline	no	no	no	no	S
ITM2009-02336	ERR4553726	F	18	22/06/2009	3+	c36	c38	yes	lineage1	lineage1.2.2.2	lineage1.2	rpoB_p.His445Asn	borderline	no	no	no	no	S
ITM2009-00804	ERR4553690	F	35	28/12/2008	2+	c37	c25	yes	lineage2	lineage2.2	lineage2.2	rpoB_p.His445Tyr	common	no	no	no	no	S
ITM2009-01969	ERR4553708	F	30	22/03/2009	2+	c37	c25	yes	lineage2	lineage2.2	lineage2.2	rpoB_p.His445Tyr	common	no	no	no	no	S
ITM2009-02580	ERR4553731	M	20	07/07/2009	2+	c37	c25	yes	lineage2	lineage2.2	lineage2.2	rpoB_p.His445Tyr	common	no	no	no	no	S
ITM2009-01739	ERR4553706	M	43	25/03/2009	3+	c38	c27	yes	lineage1	lineage1.2.2.2	lineage1.2	rpoB_p.Leu430Pro	borderline	no	no	no	no	S
ITM2009-01987	ERR4553711	M	60	26/02/2009	3+	c38	c27	yes	lineage1	lineage1.2.2.2	lineage1.2	rpoB_p.Leu430Pro	borderline	no	no	no	no	S
ITM2009-01995	ERR4553712	F	39	24/03/2009	3+	c39	c20	yes	lineage4	lineage4.1.1.1	lineage4.1	rpoB_p.Ser450Leu	common	rpoArpoC	yes	no	no	S
ITM2010-02111	ERR4553798	M	39	30/05/2010	3+	c39	c20	yes	lineage4	lineage4.1.1.1	lineage4.1	rpoB_p.Ser450Leu	common	rpoArpoC	yes	no	no	S
ITM2010-01905	ERR4553789	F	27	27/04/2010	3+	c4	c3	yes	lineage1	lineage1.2.2.2	lineage1.2	rpoB_p.Ser450Leu	common	no	no	no	no	R
ITM2007-03311	ERR4553582	F	20	02/08/2007	3+	c4	c3	yes	lineage1	lineage1.2.2.2	lineage1.2	rpoB_p.Ser450Leu	common	rpoC	yes	rpoC	yes	S
ITM2010-02091	ERR4553792	M	22	29/04/2010	2+	c4	c3	yes	lineage1	lineage1.2.2.2	lineage1.2	rpoB_p.Ser450Leu	common	rpoC	yes	rpoC	yes	R
ITM2009-02025	ERR4553716	M	22	27/04/2009	3+	c40	c21	yes	lineage4	lineage4	lineage4	rpoB_p.Ser450Leu	common	rpoC	yes	rpoC	yes	S
ITM2010-02607	ERR4553815	M	20	03/08/2010	3+	c40	c21	yes	lineage4	lineage4	lineage4	rpoB_p.Ser450Leu	common	no	no	no	no	S
ITM2009-02340	ERR4553727	F	19	21/05/2009	scanty	c41	c22	yes	lineage1	lineage1.1.3.1	lineage1.1	rpoB_p.Ser450Leu	common	no	no	no	no	S
ITM2009-02609	ERR4553736	M	39	28/05/2009	3+	c41	c22	yes	lineage1	lineage1.1.3.1	lineage1.1	rpoB_p.Ser450Leu	common	no	no	no	no	S
ITM2009-03573	ERR4553747	M	52	06/09/2009	3+	c42	c23	yes	lineage4	lineage4	lineage4	rpoB_p.Ser450Leu	common	rpoC	yes	no	no	R
ITM2010-00906	ERR4553770	M	39	24/01/2010	3+	c42	c23	yes	lineage4	lineage4	lineage4	rpoB_p.Ser450Leu	common	rpoC	yes	no	no	S
ITM2011-00911	ERR4553857	F	20	24/01/2011	3+	c42	c23	yes	lineage4	lineage4	lineage4	rpoB_p.Ser450Leu	common	rpoC	yes	no	no	S
ITM2010-02614	ERR4553818	M	15	17/08/2010	2+	c43	c28	yes	lineage3	lineage3	lineage3	rpoB_p.His445Asp	common	no	no	no	no	S
ITM2011-00963	ERR4553859	M	12	20/02/2011	2+	c43	c28	yes	lineage3	lineage3	lineage3	rpoB_p.His445Asp	common	no	no	no	no	S
ITM2010-02627	ERR4553821	M	28	10/08/2010	3+	c5	c4	yes	lineage4	lineage4	lineage4	rpoB_p.Ser450Leu	common	no	no	no	no	R
ITM2007-02225	ERR4553566	M	20	08/05/2007	2+	c5	c4	yes	lineage4	lineage4	lineage4	rpoB_p.Ser450Leu	common	no	no	no	no	S
ITM2011-00638	ERR4553856	F	21	03/01/2011	3+	c5	c4	yes	lineage4	lineage4	lineage4	rpoB_p.Ser450Leu	common	no	no	no	no	S
ITM2011-01346	ERR4553872	M	26	09/04/2011	3+	c6	c5	yes	lineage2	lineage2.2.2	lineage2.2	rpoB_p.Ser450Leu	common	no	no	no	no	R
ITM2011-01485	ERR4553877	F	25	11/05/2011	3+	c6	c5	yes	lineage2	lineage2.2.2	lineage2.2	rpoB_p.Ser450Leu	common	no	no	no	no	R
ITM2008-04282	ERR4553671	M	52	23/10/2008	3+	c6	c5	yes	lineage2	lineage2.2	lineage2.2	rpoB_p.Ser450Leu	common	rpoC	yes	rpoC	yes	S
ITM2011-01354	ERR4553874	M	61	25/04/2011	2+	c7	c6	yes	lineage4	lineage4.3.4.2	lineage4.3	rpoB_p.Ser450Leu	common	no	no	no	no	R
ITM2009-00523	ERR4553684	F	16	12/11/2008	3+	c7	c6	yes	lineage4	lineage4.3.4.2	lineage4.3	rpoB_p.Ser450Leu	common	no	no	no	no	R
ITM2005-01550	ERR4553426	M	27	05/04/2005	2+	c7	c6	yes	lineage4	lineage4.3.4.2	lineage4.3	rpoB_p.Ser450Leu	common	no	no	no	no	S
ITM2005-02418	ERR4553447	F	33	30/07/2005	3+	c7	c6	yes	lineage4	lineage4.3.4.2	lineage4.3	rpoB_p.Ser450Leu	common	no	no	no	no	S
ITM2005-02874	ERR4553458	M	20	15/09/2005	2+	c7	c6	yes	lineage4	lineage4.3.4.2	lineage4.3	rpoB_p.Ser450Leu	common	no	no	no	no	S
ITM2006-00115	ERR4553468	M	16	25/10/2005	2+	c7	c6	yes	lineage4	lineage4.3.4.2	lineage4.3	rpoB_p.Ser450Leu,rpoB_p.Glu761Asp	common	no	no	no	no	S
ITM2006-00633	ERR4553482	F	22	29/01/2006	2+	c7	c6	yes	lineage4	lineage4.3.4.2	lineage4.3	rpoB_p.Ser450Leu	common	rpoC	yes	rpoC	yes	S
ITM2006-03705	ERR4553516	F	20	11/09/2006	3+	c7	c6	yes	lineage4	lineage4.3.4.2	lineage4.3	rpoB_p.Ser450Leu	common	no	no	no	no	S
ITM2006-03883	ERR4553517	M	18	28/08/2006	3+	c7	c6	yes	lineage4	lineage4.3.4.2	lineage4.3	rpoB_p.Ser450Leu	common	no	no	no	no	S
ITM2007-01727	ERR4553544	F	21	24/01/2007	3+	c7	c6	yes	lineage4	lineage4.3.4.2	lineage4.3	rpoB_p.Ser450Leu	common	no	no	no	no	S
ITM2007-03261	ERR4553580	F	15	30/07/2007	2+	c7	c6	yes	lineage4	lineage4.3.4.2	lineage4.3	rpoB_p.Ser450Leu	common	no	no	no	no	S
ITM2008-02412	ERR4553642	F	19	24/03/2008	2+	c7	c6	yes	lineage4	lineage4.3.4.2	lineage4.3	rpoB_p.Ser450Leu	common	no	no	no	no	S
ITM2010-00365	ERR4553762	F	35	21/11/2009	3+	c7	c6	yes	lineage4	lineage4.3.4.2	lineage4.3	rpoB_p.Ser450Leu	common	no	no	no	no	S
ITM2011-00099	ERR4553833	F	50	07/10/2010	3+	c7	c6	yes	lineage4	lineage4.3.4.2	lineage4.3	rpoB_p.Ser450Leu	common	no	no	no	no	S
ITM2011-01163	ERR4553864	F	17	15/03/2011	2+	c7	c6	yes	lineage4	lineage4.3.4.2	lineage4.3	rpoB_p.Ser450Leu	common	no	no	no	no	S
ITM2011-02382	ERR4553885	M	30	23/08/2011	3+	c8	c7	yes	lineage4	lineage4.8	lineage4.8	rpoB_p.Ser450Leu	common	no	no	no	no	R
ITM2011-00375	ERR4553842	M	31	10/11/2010	3+	c8	removed	no	lineage4	lineage4.8	lineage4.8	rpoB_p.His445Asp	common	no	no	no	no	S
ITM2005-02145	ERR4553441	M	23	16/06/2005	2+	c8	c7	yes	lineage4	lineage4.8	lineage4.8	rpoB_p.Ser450Leu	common	no	no	no	no	S
ITM2005-02168	ERR4553443	F	20	02/07/2005	2+	c8	c7	yes	lineage4	lineage4.8	lineage4.8	rpoB_p.Ser450Leu	common	no	no	no	no	S
ITM2005-02880	ERR4553460	M	26	25/10/2005	2+	c8	c29	yes	lineage4	lineage4.8	lineage4.8	rpoB_p.Asp435Val	common	no	no	no	no	S
ITM2005-02896	ERR4553462	M	23	10/11/2005	3+	c8	c29	yes	lineage4	lineage4.8	lineage4.8	rpoB_p.Asp435Val	common	no	no	no	no	S
ITM2006-04070	ERR4553524	M	20	27/02/2006	2+	c8	removed	no	lineage4	lineage4.8	lineage4.8	rpoB_p.His445Tyr	common	no	no	no	no	S
ITM2008-00210	ERR4553602	M	60	12/11/2007	3+	c8	removed	no	lineage4	lineage4.8	lineage4.8	rpoB_p.Ser450Trp	common	no	no	no	no	S
ITM2008-04297	ERR4553673	M	40	21/10/2008	3+	c8	c7	yes	lineage4	lineage4.8	lineage4.8	rpoB_p.Ser450Leu	common	no	no	no	no	S
ITM2009-01725	ERR4553703	M	24	06/04/2009	2+	c8	c7	yes	lineage4	lineage4.8	lineage4.8	rpoB_p.Ser450Leu	common	no	no	no	no	S
ITM2009-02014	ERR4553714	F	18	27/04/2009	3+	c8	removed	no	lineage4	lineage4.8	lineage4.8	rpoB_p.Ile491Phe	borderline	no	no	no	no	S
ITM2009-03258	ERR4553742	F	24	05/08/2009	3+	c8	c7	yes	lineage4	lineage4.8	lineage4.8	rpoB_p.Ser450Leu	common	no	no	no	no	S
ITM2010-02405	ERR4553806	M	19	04/07/2010	2+	c8	c7	yes	lineage4	lineage4.8	lineage4.8	rpoB_p.Ser450Leu	common	no	no	no	no	S

ITM2011-00569	ERR4553851	M	40	23/12/2010	3+	c8	c7	yes	lineage4	lineage4.8	lineage4.8	rpoB_p.Ser450Leu	common	no	no	no	no	S
ITM2011-00971	ERR4553860	M	24	23/02/2011	3+	c8	c7	yes	lineage4	lineage4.8	lineage4.8	rpoB_p.Ser450Leu	common	no	no	no	no	S
ITM2007-02214	ERR4553564	F	21	07/05/2007	3+	c9	c33	yes	lineage2	lineage2.2.1	lineage2.2	rpoB_p.Asp435Tyr	borderline	no	no	no	no	R
ITM2005-02887	ERR4553461	F	20	25/10/2005	2+	c9	c33	yes	lineage2	lineage2.2.1	lineage2.2	rpoB_p.Asp435Tyr	borderline	no	no	no	no	S
ITM2010-01142	ERR4553775	M	27	15/02/2010	3+	none	none	no	lineage4	lineage4.8	lineage4.8	rpoB_p.Ser450Leu	common	no	no	no	no	R
ITM2006-00711	ERR4553486	M	36	26/01/2006	2+	none	none	no	lineage4	lineage4.8	lineage4.8	rpoB_p.His445Asp	common	no	no	no	no	S
ITM2006-04046	ERR4553519	F	21	19/09/2006	3+	none	none	no	lineage4	lineage4.8	lineage4.8	rpoB_p.Ser450Trp	common	no	no	no	no	S
ITM2007-02888	ERR4553574	M	73	06/06/2007	3+	none	none	no	lineage4	lineage4.8	lineage4.8	rpoB_p.Ser450Leu	common	no	no	no	no	S
ITM2011-00528	ERR4553846	M	51	07/12/2010	3+	none	none	no	lineage4	lineage4.8	lineage4.8	rpoB_p.His445Arg	common	no	no	no	no	S
ITM2008-03114	ERR4553650	F	26	06/07/2008	2+	none	none	no	lineage4	lineage4.9	lineage4.9	rpoB_p.His445Asp	common	no	no	no	no	R
ITM2008-04090	ERR4553668	M	25	10/08/2008	3+	none	none	no	lineage4	lineage4.9	lineage4.9	rpoB_p.His445Asp	common	no	no	no	no	R
ITM2008-02385	ERR4553641	F	26	08/04/2008	3+	none	none	no	lineage4	lineage4.9	lineage4.9	rpoB_p.His445Asp	common	no	no	no	no	S
ITM2010-02112	ERR4553799	M	30	01/06/2010	3+	none	none	no	lineage4	lineage4.9	lineage4.9	rpoB_p.His445Asp	common	no	no	no	no	S
ITM2007-02508	ERR4553571	M	32	05/06/2007	3+	none	none	no	lineage3	lineage3	lineage3	rpoB_p.Ser450Leu	common	rpoC	yes	rpoC	yes	S
ITM2008-00135	ERR4553598	M	27	09/10/2007	3+	none	none	no	lineage3	lineage3	lineage3	rpoB_p.Ser450Leu	common	rpoC	yes	rpoC	yes	S
ITM2011-01487	ERR4553878	F	25	05/05/2011	3+	none	none	no	lineage3	lineage3	lineage3	rpoB_p.Ser450Leu	common	rpoC	yes	rpoC	yes	S
ITM2008-04071	ERR4553667	F	20	10/09/2008	1+	none	none	no	lineage2	lineage2.2.1	lineage2.2	rpoB_p.Ser450Leu	common	no	no	no	no	S
ITM2006-04054	ERR4553521	M	35	08/10/2006	1+	none	none	no	lineage2	lineage2.2.1	lineage2.2	rpoB_p.Ser450Leu	common	no	no	no	no	S
ITM2007-01743	ERR4553547	M	22	15/03/2007	2+	none	none	no	lineage2	lineage2.2.1	lineage2.2	rpoB_p.Ser450Leu	common	no	no	no	no	S
ITM2009-00604	ERR4553688	F	30	19/11/2008	3+	none	none	no	lineage2	lineage2.2.1	lineage2.2	rpoB_p.Asp435Tyr	borderline	no	no	no	no	S
ITM2009-02333	ERR4553724	F	27	27/05/2009	2+	none	none	no	lineage3	lineage3	lineage3	rpoB_p.Ser450Leu	common	no	no	no	no	R
ITM2007-00264	ERR4553532	M	34	18/11/2006	3+	none	none	no	lineage3	lineage3	lineage3	rpoB_p.Ser450Leu	common	no	no	no	no	S
ITM2010-01884	ERR4553786	M	58	09/02/2010	2+	none	none	no	lineage2	lineage2.2	lineage2.2	rpoB_p.His445Tyr	common	rpoC	yes	no	no	S
ITM2006-01618	ERR4553497	M	40	27/04/2006	2+	none	none	no	lineage2	lineage2.2.1	lineage2.2	rpoB_p.Ser450Leu	common	rpoC	yes	rpoC	yes	R
ITM2009-00814	ERR4553691	M	42	01/01/2009	2+	none	none	no	lineage2	lineage2.2.1	lineage2.2	rpoB_p.Ser441Leu	common	rpoC	yes	no	no	S
ITM2006-00310	ERR4553470	M	31	22/11/2005	2+	none	none	no	lineage4	lineage4	lineage4	rpoB_p.His445Tyr	common	no	no	no	no	S
ITM2006-00603	ERR4553479	M	56	09/01/2006	2+	none	none	no	lineage1	lineage1.1.3.1	lineage1.1	rpoB_p.Leu452Pro	borderline	no	no	no	no	S
ITM2010-00873	ERR4553766	M	28	22/12/2009	3+	none	none	no	lineage1	lineage1.1.3.1	lineage1.1	rpoB_p.Ser450Leu	common	no	no	no	no	S
ITM2009-00113	ERR4553682	M	46	19/10/2008	scanty	none	none	no	lineage3	lineage3	lineage3	rpoB_p.His445Leu	borderline	rpoC	yes	no	no	S
ITM2009-02594	ERR4553734	M	35	27/07/2009	3+	none	none	no	lineage3	lineage3	lineage3	rpoB_p.Leu430Pro	borderline	rpoC	yes	no	no	S
ITM2006-01170	ERR4553495	M	45	30/03/2006	2+	none	none	no	lineage1	lineage1.2.2	lineage1.2	rpoB_c.1296_1297insTTC	common	no	no	no	no	S
ITM2011-01156	ERR4553861	M	28	08/03/2011	3+	none	none	no	lineage1	lineage1.2.2.2	lineage1.2	rpoB_c.1296_1297insTTC,rpoB_c.1299_1300insTTG	common	no	no	no	no	S
ITM2006-01602	ERR4553496	M	56	12/04/2006	3+	none	none	no	lineage1	lineage1.2.2.2	lineage1.2	rpoB_p.Ser450Leu	common	no	no	no	no	S
ITM2006-01951	ERR4553503	M	31	15/05/2006	3+	none	none	no	lineage1	lineage1.1.3.1	lineage1.1	rpoB_p.Ser450Leu	common	no	no	no	no	S
ITM2010-02110	ERR4553797	M	33	30/05/2010	3+	none	none	no	lineage1	lineage1.1.3.1	lineage1.1	rpoB_p.Ser450Leu	common	no	no	no	no	S
ITM2010-01897	ERR4553787	F	17	27/04/2010	2+	none	none	no	lineage3	lineage3	lineage3	rpoB_p.Ser450Leu	common	rpoC	yes	rpoC	yes	S
ITM2010-02618	ERR4553820	M	46	10/08/2010	3+	none	none	no	lineage1	lineage1.1.3.1	lineage1.1	rpoB_p.His445Cys	common	no	no	no	no	S
ITM2010-02400	ERR4553804	F	31	01/07/2010	2+	none	none	no	lineage2	lineage2.2.1.2	lineage2.2	rpoB_p.Ser450Leu	common	rpoC	yes	no	no	S
ITM2011-00156	ERR4553837	F	50	20/09/2010	3+	none	none	no	lineage2	lineage2.2.1	lineage2.2	rpoB_p.Ser450Leu	common	rpoC	yes	no	no	S
ITM2006-00583	ERR4553478	M	33	21/12/2005	3+	none	none	no	lineage4	lineage4	lineage4	rpoB_p.Val170Phe	common	rpoC	yes	no	no	S
ITM2009-02315	ERR4553720	M	52	15/06/2009	1+	none	none	no	lineage4	lineage4	lineage4	rpoB_p.His445Asp	common	no	no	no	no	S
ITM2009-01130	ERR4553695	M	20	03/02/2009	3+	none	none	no	lineage4	lineage4.3.4.2	lineage4.3	rpoB_p.Ser450Leu	common	no	no	no	no	S
ITM2007-02201	ERR4553563	M	36	11/04/2007	2+	none	none	no	lineage4	lineage4.3.4.2	lineage4.3	rpoB_p.Asp435Val	common	no	no	no	no	S
ITM2007-03603	ERR4553593	M	21	17/09/2007	3+	none	none	no	lineage4	lineage4.3.4.2	lineage4.3	rpoB_p.Ser450Leu	common	no	no	no	no	S
ITM2008-01305	ERR4553616	M	31	10/01/2008	1+	none	none	no	lineage4	lineage4.3.4.2	lineage4.3	rpoB_p.Ser450Leu	common	no	no	no	no	S
ITM2010-00228	ERR4553756	M	25	07/10/2009	2+	none	none	no	lineage4	lineage4.3.4.2	lineage4.3	rpoB_p.Ser450Leu	common	rpoC	yes	rpoC	yes	S
ITM2011-01501	ERR4553879	F	25	12/05/2011	3+	none	none	no	lineage4	lineage4.1.1.1	lineage4.1	rpoB_p.Ser450Leu	common	no	no	no	no	R
ITM2009-00497	ERR4553683	M	27	08/11/2008	3+	none	none	no	lineage4	lineage4.1.1.1	lineage4.1	rpoB_p.Ser450Leu	common	no	no	no	no	S
ITM2006-00699	ERR4553485	M	23	04/01/2006	2+	none	none	no	lineage4	lineage4.1.1.1	lineage4.1	rpoB_p.Ser450Leu	common	rpoA	yes	no	no	S
ITM2008-00219	ERR4553604	F	22	27/11/2007	2+	none	none	no	lineage4	lineage4.1.1.1	lineage4.1	rpoB_p.Ser450Leu	common	rpoA	yes	no	no	S
ITM2010-02417	ERR4553810	M	20	24/07/2010	3+	none	none	no	lineage4	lineage4.1.1.1	lineage4.1	rpoB_p.Ser450Leu	common	rpoA	yes	no	no	S
ITM2011-00632	ERR4553855	M	23	05/01/2011	3+	none	none	no	lineage4	lineage4.1.1.1	lineage4.1	rpoB_p.Ser450Leu	common	rpoA	yes	no	no	R
ITM2008-03148	ERR4553656	M	50	08/07/2008	3+	none	none	no	lineage2	lineage2.2.1	lineage2.2	rpoB_p.Asp435Tyr	borderline	no	no	no	no	S
ITM2010-00244	ERR4553758	M	37	01/11/2009	1+	none	none	no	lineage2	lineage2.2.1	lineage2.2	rpoB_p.His445Tyr	common	no	no	no	no	S
ITM2010-01407	ERR4553777	F	23	01/03/2010	3+	none	none	no	lineage2	lineage2.2.1	lineage2.2	rpoB_p.Ser450Leu	common	no	no	no	no	S
ITM2010-02130	ERR4553800	F	45	16/06/2010	2+	none	none	no	lineage2	lineage2.2.1	lineage2.2	rpoB_p.Ser450Leu	common	rpoC	yes	rpoC	yes	S
ITM2011-00170	ERR4553838	M	61	29/09/2010	3+	none	none	no	lineage2	lineage2.2.1	lineage2.2	rpoB_p.His445Tyr	common	no	no	no	no	S
ITM2011-00604	ERR4553853	M	26	26/12/2010	3+	none	none	no	lineage2	lineage2.2.1	lineage2.2	rpoB_p.His445Leu	borderline	no	no	no	no	S
ITM2010-01661	ERR4553785	M	25	11/04/2010	2+	none	none	no	lineage1	lineage1.1.3.1	lineage1.1	rpoB_p.His445Tyr	common	no	no	no	no	R
ITM2010-02106	ERR4553795	M	50	15/05/2010	3+	none	none	no	lineage3	lineage3	lineage3	rpoB_p.Ser450Leu	common	no	no	no	no	R
ITM2010-02808	ERR4553827	F	38	22/09/2010	3+	none	none	no	lineage2	lineage2.2.1	lineage2.2	rpoB_p.Ser450Leu	common	no	no	no	no	R
ITM2011-00113	ERR4553834	F	23	03/10/2010	2+	none	none	no	lineage4	lineage4	lineage4	rpoB_p.Ser450Leu	common	no	no	no	no	R
ITM2011-01331	ERR4553870	F	37	24/03/2011	3+	none	none	no	lineage2	lineage2.2.1	lineage2.2	rpoB_p.Ser450Val,rpoB_p.Leu430Arg	inferred	no	no	no	no	R
ITM2009-02009	ERR4553713	M	15	04/05/2009	2+	none	none	no	lineage4	lineage4.8	lineage4.8	rpoB_p.Ser450Leu	common	no	no	no	no	S
ITM2006-02368	ERR4553507	F	52	12/06/2006	2+	none	none	no	lineage4	lineage4.3.4.2	lineage4.3	rpoB_p.His445Asp	common	no	no	no	no	R
ITM2008-03113	ERR4553649	M	65	30/06/2008	3+	none	none	no	lineage3	lineage3	lineage3	rpoB_p.Ser450Leu	common	no	no	no	no	R
ITM2009-00101	ERR4553680	M	50	30/10/2008	2+	none	none	no	lineage4	lineage4.1.1.3	lineage4.1	rpoB_p.Ser450Leu	common	no	no	no	no	R
ITM2009-01126	ERR4553694	M	36	15/01/2009	1+	none	none	no	lineage2	lineage2.2.1	lineage2.2	rpoB_p.Ser450Leu	common	no	no	no	no	R
ITM2009-01980	ERR4553710	F	21	16/02/2009	3+	none	none	no	lineage3	lineage3.1.2.1	lineage3.1	rpoB_p.Ser450Leu	common	no	no	no	no	R
ITM2005-00677	ERR4553421	M	40	06/01/2005	1+	none	none	no	lineage3	lineage3	lineage3	rpoB_p.Ser450Leu	common	no	no	no	no	S
ITM2005-00822	ERR4553422	F	18	05/02/2005	1+	none	none	no	lineage1	lineage1.1.3.1	lineage1.1	rpoB_p.Asp435Val	common	no	no	no	no	S
ITM2005-00840	ERR4553423	F	46	12/01/2005	3+	none	none	no	lineage1	lineage1.2.2.2	lineage1.2	rpoB_p.Ser450Leu	common	no	no	no	no	S
ITM2005-01434	ERR4553424	F	46	03/03/2005	1+	none	none	no	lineage2	lineage2.2.1	lineage2.2	rpoB_p.Asn437Asp,rpoB_p.Ser431Arg,rpoB_p.Asp435Gly	inferred	no	no	rpoB	yes	S
ITM2005-01831	ERR4553431	M	33	07/04/2005	3+	none	none	no	lineage3	lineage3	lineage3	rpoB_p.Ser450Leu	common	rpoC	yes	no	no	S
ITM2005-01866	ERR4553433	M	38	16/05/2005	3+	none	none	no	lineage1	lineage1.2.2.2	lineage1.2	rpoB_p.Ser450Leu	common	rpoC	yes	no	no	S
ITM2005-01873	ERR4553434	F	20	16/04/2005	1+	none	none	no	lineage2	lineage2.2.1	lineage2.2	rpoB_p.Val170Phe	common	rpoC	yes	no	no	S
ITM2005-01898	ERR4553435	M	60	09/05/2005	3+	none	none	no	lineage1	lineage1.1.3.1	lineage1.1	rpoB_p.Ser450Leu	common	rpoC	yes	no	no	S
ITM2005-02090	ERR4553437	F	35	13/06/2005	2+	none	none	no	lineage1	lineage1.1.2	lineage1.1	rpoB_p.Ser450Leu	common	no	no	no	no	S
ITM2005-02195	ERR4553445	M	43	03/07/2005	1+	none	none	no	lineage3	lineage3	lineage3	rpoB_p.Ser450Leu	common	rpoC	yes	rpoC	yes	S
ITM2005-02669	ERR4553449	F	20	02/08/2005	2+	none	none	no	lineage3	lineage3	lineage3	rpoB_p.Ser450Leu	common	no	no	no	no	S
ITM2005-02675	ERR4553450	M	46	31/08/2005	3+	none	none	no	lineage2	lineage2.2.1	lineage2.2	rpoB_p.Gln432Pro	common	no	no	no	no	S
ITM2005-02692	ERR4553452	M	43	22/08/2005	1+	none	none	no	lineage1	lineage1.1.3.1	lineage1.1	rpoB_p.His445Asp	common	no	no	no	no	S
ITM2005-02712	ERR4553453	M	50	20/08/2005	3+	none	none	no	lineage3	lineage3	lineage3	rpoB_p.Ser450Leu	common	no	no	no	no	S
ITM2005-02784	ERR4553455	F	33	25/09/2005	2+	none	none	no	lineage3	lineage3.1	lineage3.1	rpoB_p.Ser450Leu	common	rpoC	yes	no	no	S

ITM2005-02924	ERR4553464	M	36	11/09/2005	3+	none	none	no	lineage1	lineage1.1.3.1	lineage1.1	rpoB_p.Leu452Pro	borderline	no	no	no	no	S
ITM2005-02936	ERR4553465	M	28	10/10/2005	2+	none	none	no	lineage1	lineage1.2.2	lineage1.2	rpoB_p.Ser450Leu	common	no	no	no	no	S
ITM2006-00287	ERR4553469	M	29	11/12/2005	3+	none	none	no	lineage1	lineage1.1.3.1	lineage1.1	rpoB_p.Ser450Leu	common	no	no	no	no	S
ITM2006-00339	ERR4553473	M	50	08/11/2005	2+	none	none	no	lineage1	lineage1.1.3	lineage1.1	rpoB_p.Ser450Leu	common	rpoC	yes	rpoC	yes	S
ITM2006-00342	ERR4553474	F	20	29/11/2005	1+	none	none	no	lineage4	lineage4.3	lineage4.3	rpoB_p.Ser450Leu	common	rpoC	yes	no	no	S
ITM2006-00344	ERR4553475	M	60	30/11/2005	2+	none	none	no	lineage4	lineage4.9	lineage4.9	rpoB_p.Ser450Trp	common	no	no	no	no	S
ITM2006-00352	ERR4553476	M	60	07/12/2005	2+	none	none	no	lineage3	lineage3	lineage3	rpoB_p.Ser450Leu	common	rpoC	yes	no	no	S
ITM2006-00663	ERR4553484	M	43	04/01/2006	3+	none	none	no	lineage1	lineage1.1.3.1	lineage1.1	rpoB_p.Leu452Pro	borderline	no	no	no	no	S
ITM2006-00733	ERR4553487	M	44	18/01/2006	2+	none	none	no	lineage3	lineage3.1.2.1	lineage3.1	rpoB_p.His445Gln,rpoB_p.Ser450Trp	inferred	no	no	no	no	S
ITM2006-00741	ERR4553488	F	30	05/01/2006	3+	none	none	no	lineage4	lineage4.8	lineage4.8	rpoB_p.Asp435Tyr	borderline	no	no	no	no	S
ITM2006-01152	ERR4553493	M	52	07/02/2006	2+	none	none	no	lineage1	lineage1.1.3.1	lineage1.1	rpoB_p.Ser450Trp	common	no	no	no	no	S
ITM2006-01936	ERR4553499	M	56	27/04/2006	2+	none	none	no	lineage1	lineage1.2.2	lineage1.2	rpoB_p.Ser450Leu	common	no	no	no	no	S
ITM2006-01937	ERR4553500	M	35	10/05/2006	2+	none	none	no	lineage2	lineage2.2.1	lineage2.2	rpoB_p.Ser450Leu	common	no	no	no	no	S
ITM2006-01941	ERR4553501	M	69	21/05/2006	3+	none	none	no	lineage1	lineage1.2.2.2	lineage1.2	rpoB_p.Ile491Phe	borderline	no	no	no	no	S
ITM2006-02361	ERR4553504	M	31	15/05/2006	3+	none	none	no	lineage1	lineage1.1.3.3	lineage1.1	rpoB_p.Ser450Leu	common	no	no	no	no	S
ITM2006-02362	ERR4553505	M	45	15/05/2006	3+	none	none	no	lineage1	lineage1.1.3.1	lineage1.1	rpoB_p.His445Tyr	common	no	no	no	no	S
ITM2006-02387	ERR4553509	M	24	13/06/2006	2+	none	none	no	lineage1	lineage1.1.3.1	lineage1.1	rpoB_p.His445Asp	common	no	no	no	no	S
ITM2006-03446	ERR4553514	M	24	27/07/2006	2+	none	none	no	lineage4	lineage4.8	lineage4.8	rpoB_p.Ser450Leu	common	rpoC	yes	no	no	S
ITM2006-03902	ERR4553518	F	26	14/08/2006	1+	none	none	no	lineage1	lineage1.2.2	lineage1.2	rpoB_p.Ser450Leu	common	rpoC	yes	rpoC	yes	S
ITM2007-00253	ERR4553528	M	26	15/11/2006	2+	none	none	no	lineage2	lineage2.2.1	lineage2.2	rpoB_p.Gln432Lys	common	no	no	no	no	S
ITM2007-00258	ERR4553529	F	20	22/11/2006	3+	none	none	no	lineage4	lineage4.1.2.1	lineage4.1	rpoB_p.His445Asp	common	no	no	no	no	S
ITM2007-00260	ERR4553530	M	56	18/11/2006	3+	none	none	no	lineage1	lineage1.1.3.1	lineage1.1	rpoB_p.Asp435Tyr	borderline	no	no	no	no	S
ITM2007-00277	ERR4553533	M	22	09/12/2006	2+	none	none	no	lineage2	lineage2.2.1	lineage2.2	rpoB_p.His445Tyr	common	no	no	no	no	S
ITM2007-01344	ERR4553537	M	46	03/02/2007	3+	none	none	no	lineage1	lineage1.2.2	lineage1.2	rpoB_p.His445Tyr	common	no	no	no	no	S
ITM2007-01356	ERR4553539	M	28	06/07/2007	2+	none	none	no	lineage2	lineage2.2.1	lineage2.2	rpoB_p.Ser450Leu	common	rpoC	yes	no	no	S
ITM2007-01369	ERR4553540	M	37	25/02/2007	3+	none	none	no	lineage3	lineage3	lineage3	rpoB_p.His445Leu	borderline	no	no	no	no	S
ITM2007-01725	ERR4553543	M	52	04/03/2007	2+	none	none	no	lineage2	lineage2.2.1	lineage2.2	rpoB_p.His445Tyr	common	no	no	no	no	S
ITM2007-01737	ERR4553546	M	20	25/02/2007	2+	none	none	no	lineage4	lineage4	lineage4	rpoB_p.Ser450Leu	common	no	no	no	no	S
ITM2007-01767	ERR4553549	M	24	06/03/2007	3+	none	none	no	lineage3	lineage3.1.2.1	lineage3.1	rpoB_p.Asp435Val	common	no	no	no	no	S
ITM2007-01855	ERR4553553	M	32	19/03/2007	1+	none	none	no	lineage1	lineage1.2.2.2	lineage1.2	rpoB_p.Ser450Leu	common	rpoC	yes	no	no	S
ITM2007-01857	ERR4553554	M	26	08/04/2007	scanty	none	none	no	lineage3	lineage3	lineage3	rpoB_p.Ser450Leu	common	no	no	no	no	S
ITM2007-01870	ERR4553556	M	51	18/04/2007	3+	none	none	no	lineage3	lineage3	lineage3	rpoB_p.Ser450Leu	common	no	no	no	no	S
ITM2007-01890	ERR4553558	M	66	08/04/2007	3+	none	none	no	lineage1	lineage1.2.2.2	lineage1.2	rpoB_p.His445Asn	borderline	no	no	no	no	S
ITM2007-01891	ERR4553559	M	27	18/04/2007	2+	none	none	no	lineage3	lineage3	lineage3	rpoB_p.Asp435Val	common	no	no	no	no	S
ITM2007-02243	ERR4553568	M	30	21/05/2007	3+	none	none	no	lineage4	lineage4	lineage4	rpoB_p.Ser450Leu,rpoB_p.Ile480Val	common	no	no	no	no	S
ITM2007-02874	ERR4553573	M	40	02/07/2007	2+	none	none	no	lineage1	lineage1.1.3.1	lineage1.1	rpoB_p.Ser450Leu	common	rpoC	yes	rpoC	yes	S
ITM2007-02925	ERR4553575	M	45	18/07/2007	2+	none	none	no	lineage2	lineage2.2.1.1	lineage2.2	rpoB_p.Asp435Val	common	no	no	no	no	S
ITM2007-03300	ERR4553581	M	27	21/08/2007	3+	none	none	no	lineage1	lineage1.2.2.2	lineage1.2	rpoB_p.His445Tyr	common	rpoC	yes	no	no	S
ITM2007-03324	ERR4553583	M	48	16/08/2007	3+	none	none	no	lineage2	lineage2.2.1	lineage2.2	rpoB_p.His445Asp	common	no	no	no	no	S
ITM2007-03326	ERR4553584	F	46	22/08/2007	2+	none	none	no	lineage1	lineage1.2.2.2	lineage1.2	rpoB_p.Ile491Phe	borderline	rpoC	yes	no	no	S
ITM2007-03521	ERR4553587	M	55	26/08/2007	3+	none	none	no	lineage1	lineage1.1.3.1	lineage1.1	rpoB_p.His445Leu	borderline	no	no	no	no	S
ITM2007-03592	ERR4553591	M	34	23/08/2007	3+	none	none	no	lineage4	lineage4.8	lineage4.8	rpoB_p.Ser450Leu	common	no	no	no	no	S
ITM2007-03593	ERR4553592	M	60	28/08/2007	3+	none	none	no	lineage1	lineage1.1.3.1	lineage1.1	rpoB_p.Ile491Phe	borderline	no	no	no	no	S
ITM2007-03621	ERR4553594	M	44	25/08/2007	3+	none	none	no	lineage1	lineage1.1.3.1	lineage1.1	rpoB_p.His445Asp	common	no	no	no	no	S
ITM2008-00131	ERR4553597	M	55	17/10/2007	3+	none	none	no	lineage1	lineage1.2.2.2	lineage1.2	rpoB_p.His445Tyr	common	no	no	no	no	S
ITM2008-00141	ERR4553599	M	35	02/10/2007	2+	none	none	no	lineage4	lineage4.4.1.1	lineage4.4	rpoB_p.Gln432Pro	common	no	no	no	no	S
ITM2008-00142	ERR4553600	M	20	07/10/2007	3+	none	none	no	lineage1	lineage1.1.2	lineage1.1	rpoB_p.His445Tyr	common	no	no	no	no	S
ITM2008-00207	ERR4553601	M	30	11/11/2007	1+	none	none	no	lineage1	lineage1.1.3.1	lineage1.1	rpoB_p.Ser450Leu	common	rpoC	yes	no	no	S
ITM2008-00243	ERR4553605	M	40	18/11/2007	2+	none	none	no	lineage1	lineage1.1.3.3	lineage1.1	rpoB_p.Ser450Leu	common	no	no	no	no	R
ITM2008-00247	ERR4553606	F	27	18/10/2007	3+	none	none	no	lineage2	lineage2.2.1	lineage2.2	rpoB_p.Ser450Leu	common	rpoC	yes	no	no	S
ITM2008-00647	ERR4553609	M	43	19/11/2007	3+	none	none	no	lineage3	lineage3	lineage3	rpoB_p.Ser450Leu	common	no	no	no	no	S
ITM2008-00678	ERR4553611	F	35	26/11/2007	2+	none	none	no	lineage3	lineage3.1	lineage3.1	rpoB_p.Ser450Leu	common	no	no	no	no	S
ITM2008-00688	ERR4553612	M	48	09/12/2007	3+	none	none	no	lineage1	lineage1.1.3.1	lineage1.1	rpoB_p.His445Gly	inferred	rpoC	yes	no	no	S
ITM2008-01048	ERR4553615	F	20	10/12/2007	3+	none	none	no	lineage1	lineage1.1.3.1	lineage1.1	rpoB_p.Ser450Leu	common	no	no	no	no	S
ITM2008-01307	ERR4553617	M	51	13/01/2008	2+	none	none	no	lineage1	lineage1.1.2	lineage1.1	rpoB_p.Ser450Leu,rpoB_p.Ala286Val	common	rpoC	yes	no	no	S
ITM2008-01331	ERR4553621	M	25	08/01/2008	3+	none	none	no	lineage3	lineage3	lineage3	rpoB_p.Ser450Leu	common	no	no	no	no	S
ITM2008-01544	ERR4553622	M	30	12/11/2007	1+	none	none	no	lineage1	lineage1.1.3	lineage1.1	rpoB_p.Ser450Leu	common	no	no	no	no	S
ITM2008-01547	ERR4553623	M	20	13/11/2007	2+	none	none	no	lineage2	lineage2.2	lineage2.2	rpoB_p.Ser450Leu	common	no	no	no	no	R
ITM2008-01717	ERR4553626	M	61	11/12/2007	3+	none	none	no	lineage3	lineage3.1	lineage3.1	rpoB_p.Ser450Leu	common	rpoC	yes	rpoC	yes	S
ITM2008-01770	ERR4553627	F	20	04/11/2007	3+	none	none	no	lineage4	lineage4	lineage4	rpoB_p.Ser450Trp	common	no	no	no	no	S
ITM2008-01773	ERR4553628	M	31	10/01/2008	1+	none	none	no	lineage1	lineage1.1.3	lineage1.1	rpoB_p.Ser450Leu	common	no	no	no	no	S
ITM2008-01807	ERR4553631	M	19	09/02/2008	3+	none	none	no	lineage2	lineage2.2.1	lineage2.2	rpoB_p.Ser450Leu	common	no	no	no	no	S
ITM2008-01811	ERR4553632	F	35	09/01/2008	3+	none	none	no	lineage1	lineage1.1.3.1	lineage1.1	rpoB_p.His445Tyr	common	no	no	no	no	S
ITM2008-01822	ERR4553633	M	46	07/01/2008	3+	none	none	no	lineage4	lineage4	lineage4	rpoB_p.Ser450Leu	common	no	no	no	no	S
ITM2008-02031	ERR4553635	F	37	09/03/2008	2+	none	none	no	lineage2	lineage2.2	lineage2.2	rpoB_p.His445Asp,rpoB_p.Asp435Gly	inferred	no	no	no	no	S
ITM2008-02380	ERR4553640	F	29	27/04/2008	3+	none	none	no	lineage1	lineage1.2.2	lineage1.2	rpoB_p.Ser450Leu	common	no	no	no	no	S
ITM2008-02737	ERR4553643	M	35	13/05/2008	3+	none	none	no	lineage1	lineage1.2.2.2	lineage1.2	rpoB_p.His445Asp	common	no	no	no	no	S
ITM2008-02755	ERR4553646	M	18	08/04/2008	3+	none	none	no	lineage3	lineage3	lineage3	rpoB_p.Leu452Pro	borderline	no	no	no	no	S
ITM2008-03103	ERR4553648	M	55	18/05/2008	2+	none	none	no	lineage1	lineage1.1.3.1	lineage1.1	rpoB_p.Val170Phe	common	no	no	rpoB	yes	S
ITM2008-03131	ERR4553652	M	33	05/06/2008	3+	none	none	no	lineage2	lineage2.2.1	lineage2.2	rpoB_p.Asp435Tyr	borderline	no	no	no	no	S
ITM2008-03342	ERR4553657	F	30	08/07/2008	2+	none	none	no	lineage3	lineage3.1.2.1	lineage3.1	rpoB_p.Ser450Leu	common	no	no	no	no	S
ITM2008-03352	ERR4553658	M	23	14/07/2008	3+	none	none	no	lineage4	lineage4.9	lineage4.9	rpoB_p.Asp435Tyr	borderline	no	no	no	no	S
ITM2008-04059	ERR4553666	M	25	14/09/2008	3+	none	none	no	lineage3	lineage3	lineage3	rpoB_p.Ser450Leu	common	rpoC	yes	no	no	S
ITM2008-04284	ERR4553672	M	24	25/10/2008	3+	none	none	no	lineage3	lineage3	lineage3	rpoB_p.Leu430Pro	borderline	no	no	no	no	S
ITM2008-04310	ERR4553676	M	35	25/10/2008	3+	none	none	no	lineage2	lineage2.2.1	lineage2.2	rpoB_p.His445Tyr	common	no	no	no	no	S
ITM2008-04316	ERR4553677	M	36	28/09/2008	2+	none	none	no	lineage3	lineage3.1.2.1	lineage3.1	rpoB_p.Asp435Val	common	no	no	no	no	S
ITM2009-00548	ERR4553685	M	40	16/10/2008	3+	none	none	no	lineage1	lineage1.1.2	lineage1.1	rpoB_c.1296_1297insTTC	common	no	no	no	no	S
ITM2009-00549	ERR4553686	M	41	21/09/2008	2+	none	none	no	lineage1	lineage1.1.3.1	lineage1.1	rpoB_p.His445Tyr	common	no	no	no	no	S
ITM2009-00602	ERR4553687	M	66	24/10/2008	2+	none	none	no	lineage3	lineage3	lineage3	rpoB_p.Ser450Trp	common	no	no	no	no	S
ITM2009-01137	ERR4553696	M	45	15/02/2009	2+	none	none	no	lineage4	lineage4.8	lineage4.8	rpoB_p.Ser450Leu	common	no	no	no	no	S
ITM2009-01144	ERR4553698	M	30	26/02/2009	3+	none	none	no	lineage1	lineage1.1.3.1	lineage1.1	rpoB_p.His445Tyr	common	no	no	no	no	S
ITM2009-01711	ERR4553699	M	18	21/04/2009	scanty	none	none	no	lineage3	lineage3.1.2	lineage3.1	rpoB_p.Ser450Leu	common	rpoC	yes	rpoC	yes	S
ITM2009-01712	ERR4553700	M	45	21/04/2009	3+	none	none	no	lineage4	lineage4.1.1.1	lineage4.1	rpoB_p.Ser450Trp	common	rpoA	yes	no	no	S
ITM2009-01716	ERR4553701	M	57	01/04/2009	3+	none	none	no	lineage3	lineage3	lineage3	rpoB_p.His445Arg	common	no	no	no	no	S

ITM2009-01726	ERR4553704	F	27	16/04/2009	1+	none	none	no	lineage3	lineage3	lineage3	rpoB_p.Ser450Leu	common	no	no	rpoB	yes	S
ITM2009-01967	ERR4553707	M	28	02/03/2009	3+	none	none	no	lineage1	lineage1.1.3.1	lineage1.1	rpoB_p.Asp435Val	common	no	no	no	no	S
ITM2009-01970	ERR4553709	F	36	22/03/2009	scanty	none	none	no	lineage4	lineage4.8	lineage4.8	rpoB_p.Asp435Val	common	no	no	no	no	S
ITM2009-02314	ERR4553719	M	30	11/06/2009	2+	none	none	no	lineage1	lineage1.1.3.1	lineage1.1	rpoB_p.Ser450Leu	common	rpoC	yes	rpoC	yes	S
ITM2009-02319	ERR4553721	M	52	29/06/2009	3+	none	none	no	lineage4	lineage4	lineage4	rpoB_p.Val170Phe	common	no	no	no	no	S
ITM2009-02325	ERR4553722	M	37	26/05/2009	3+	none	none	no	lineage4	lineage4.8	lineage4.8	rpoB_p.Ser450Leu	common	rpoC	yes	no	no	S
ITM2009-02361	ERR4553729	F	25	02/06/2009	3+	none	none	no	lineage2	lineage2.2.1	lineage2.2	rpoB_p.Leu430Pro,rpoB_p.Ser493Leu	borderline	no	no	no	no	R
ITM2009-02577	ERR4553730	F	35	23/06/2009	3+	none	none	no	lineage1	lineage1.2.2	lineage1.2	rpoB_p.His445Asp	common	no	no	no	no	S
ITM2009-02581	ERR4553732	M	21	16/07/2009	2+	none	none	no	lineage1	lineage1.2.2.2	lineage1.2	rpoB_p.Ser450Leu	common	rpoC	yes	no	no	S
ITM2009-02618	ERR4553737	M	50	20/06/2009	2+	none	none	no	lineage2	lineage2.2.1	lineage2.2	rpoB_p.Ser450Leu	common	no	no	no	no	S
ITM2009-03234	ERR4553738	M	26	12/07/2009	1+	none	none	no	lineage4	lineage4.1.1.1	lineage4.1	rpoB_p.His445Tyr	common	rpoA	yes	no	no	S
ITM2009-03251	ERR4553739	F	51	22/07/2009	3+	none	none	no	lineage1	lineage1.1.2	lineage1.1	rpoB_p.Leu452Pro	borderline	rpoC	yes	no	no	S
ITM2009-03254	ERR4553740	F	27	30/07/2009	3+	none	none	no	lineage1	lineage1.1.3.3	lineage1.1	rpoB_p.Ser450Trp	common	rpoC	yes	rpoC	yes	S
ITM2009-03255	ERR4553741	M	60	02/08/2009	2+	none	none	no	lineage1	lineage1.1.3.1	lineage1.1	rpoB_p.Leu452Pro	borderline	no	no	no	no	S
ITM2009-03262	ERR4553743	M	32	08/08/2009	3+	none	none	no	lineage4	lineage4.8	lineage4.8	rpoB_p.Ser450Leu	common	rpoC	yes	no	no	S
ITM2009-03761	ERR4553751	M	50	15/09/2009	2+	none	none	no	lineage1	lineage1.2.2.2	lineage1.2	rpoB_p.His445Pro	inferred	no	no	no	no	S
ITM2009-03763	ERR4553752	F	28	30/09/2009	3+	none	none	no	lineage4	lineage4.5	lineage4.5	rpoB_p.Ser450Leu	common	rpoC	yes	rpoC	yes	S
ITM2009-03773	ERR4553754	M	40	04/10/2009	3+	none	none	no	lineage1	lineage1.2.2.2	lineage1.2	rpoB_p.Ser450Leu	common	rpoC	yes	no	no	S
ITM2009-03770	ERR4553753	M	46	26/09/2009	3+	none	none	no	lineage3	lineage3	lineage3	rpoB_p.His445Asp	common	no	no	no	no	S
ITM2010-00234	ERR4553757	F	35	02/11/2009	3+	none	none	no	lineage1	lineage1.1.3.1	lineage1.1	rpoB_p.Ser450Trp	common	rpoC	yes	no	no	S
ITM2010-00360	ERR4553760	M	26	21/10/2009	1+	none	none	no	lineage1	lineage1.1.3	lineage1.1	rpoB_p.Ser450Leu	common	no	no	no	no	S
ITM2010-00363	ERR4553761	M	29	17/11/2009	3+	none	none	no	lineage1	lineage1.2.2.2	lineage1.2	rpoB_p.His445Tyr	common	no	no	no	no	S
ITM2010-01117	ERR4553771	F	23	18/01/2010	2+	none	none	no	lineage1	lineage1.2.2.2	lineage1.2	rpoB_p.Leu452Pro	borderline	no	no	no	no	S
ITM2010-01122	ERR4553772	M	43	01/02/2010	3+	none	none	no	lineage1	lineage1.2.2.2	lineage1.2	rpoB_p.Ser450Trp	common	no	no	no	no	S
ITM2010-01138	ERR4553773	F	13	15/02/2010	2+	none	none	no	lineage2	lineage2.2.1	lineage2.2	rpoB_p.Ser450Leu	common	no	no	no	no	S
ITM2010-01403	ERR4553776	M	54	22/02/2010	2+	none	none	no	lineage1	lineage1.1.2	lineage1.1	rpoB_p.Ser450Leu	common	no	no	no	no	S
ITM2010-01424	ERR4553779	M	47	22/03/2010	2+	none	none	no	lineage1	lineage1.1.3.1	lineage1.1	rpoB_p.His445Cys	common	no	no	no	no	S
ITM2010-01644	ERR4553781	M	30	22/03/2010	3+	none	none	no	lineage1	lineage1.2.2.2	lineage1.2	rpoB_p.His445Asp	common	no	no	no	no	S
ITM2010-01651	ERR4553782	M	42	23/03/2010	2+	none	none	no	lineage1	lineage1.1.3	lineage1.1	rpoB_p.Ser450Leu	common	no	no	no	no	S
ITM2010-01654	ERR4553783	M	62	12/04/2010	2+	none	none	no	lineage1	lineage1.1.3.1	lineage1.1	rpoB_p.Asp435Val	common	no	no	no	no	S
ITM2010-01658	ERR4553784	M	41	08/04/2010	3+	none	none	no	lineage4	lineage4.8	lineage4.8	rpoB_p.Ser450Leu	common	no	no	no	no	S
ITM2010-01901	ERR4553788	M	37	11/04/2010	3+	none	none	no	lineage1	lineage1.2.2.2	lineage1.2	rpoB_p.His445Asp	common	rpoC	yes	no	no	S
ITM2010-02100	ERR4553794	M	25	25/05/2010	2+	none	none	no	lineage2	lineage2.2.1	lineage2.2	rpoB_p.Asp435Val	common	no	no	no	no	S
ITM2010-02109	ERR4553796	F	20	30/05/2010	3+	none	none	no	lineage2	lineage2.2.1	lineage2.2	rpoB_p.Asp435Val	common	rpoC	yes	no	no	S
ITM2010-02131	ERR4553801	M	76	14/06/2010	2+	none	none	no	lineage1	lineage1.2.2	lineage1.2	rpoB_p.His445Tyr	common	no	no	no	no	S
ITM2010-02385	ERR4553802	F	26	09/06/2010	2+	none	none	no	lineage2	lineage2.2.1	lineage2.2	rpoB_p.Ser450Leu	common	no	no	no	no	S
ITM2010-02404	ERR4553805	M	38	04/07/2010	3+	none	none	no	lineage1	lineage1.1.3.1	lineage1.1	rpoB_p.Ser450Leu	common	no	no	no	no	S
ITM2010-02409	ERR4553808	M	50	06/07/2010	3+	none	none	no	lineage1	lineage1.1.3.1	lineage1.1	rpoB_p.Ser450Leu	common	no	no	no	no	S
ITM2010-02592	ERR4553812	M	36	05/08/2010	3+	none	none	no	lineage4	lineage4.9	lineage4.9	rpoB_p.Ser450Trp	common	no	no	no	no	S
ITM2010-02598	ERR4553813	M	18	02/08/2010	3+	none	none	no	lineage4	lineage4.3.4.2	lineage4.3	rpoB_p.Ser450Leu	common	no	no	no	no	S
ITM2010-02599	ERR4553814	F	22	03/08/2010	3+	none	none	no	lineage1	lineage1.2.2.2	lineage1.2	rpoB_p.His445Asn,rpoB_p.Asp435Glu	borderline	no	no	no	no	S
ITM2010-02615	ERR4553819	M	25	13/07/2010	1+	none	none	no	lineage3	lineage3	lineage3	rpoB_p.Ser450Leu	common	rpoC	yes	no	no	S
ITM2010-02763	ERR4553822	M	67	02/08/2010	3+	none	none	no	lineage1	lineage1.1.2	lineage1.1	rpoB_p.Gln432Arg	inferred	rpoA	yes	rpoA	yes	S
ITM2010-02799	ERR4553824	F	22	09/09/2010	3+	none	none	no	lineage4	lineage4	lineage4	rpoB_p.Ser450Leu	common	no	no	no	no	S
ITM2010-02806	ERR4553826	M	51	15/09/2010	2+	none	none	no	lineage2	lineage2.2.1	lineage2.2	rpoB_p.Ser450Leu	common	rpoC	yes	rpoC	yes	S
ITM2010-02816	ERR4553828	F	20	27/09/2010	3+	none	none	no	lineage2	lineage2.2.1	lineage2.2	rpoB_p.His445Tyr	common	no	no	no	no	S
ITM2011-00068	ERR4553830	M	20	03/10/2010	3+	none	none	no	lineage4	lineage4	lineage4	rpoB_p.Ser450Leu	common	no	no	no	no	S
ITM2011-00127	ERR4553836	F	20	20/10/2010	3+	none	none	no	lineage3	lineage3.1	lineage3.1	rpoB_p.Ser450Leu	common	rpoC	yes	no	no	S
ITM2011-00383	ERR4553843	M	55	10/11/2010	3+	none	none	no	lineage1	lineage1.1.3.1	lineage1.1	rpoB_p.His445Arg	common	rpoC	yes	no	no	S
ITM2011-00518	ERR4553845	M	26	02/12/2010	2+	none	none	no	lineage3	lineage3	lineage3	rpoB_p.His445Asp	common	no	no	no	no	S
ITM2011-00534	ERR4553847	M	60	09/12/2010	3+	none	none	no	lineage3	lineage3	lineage3	rpoB_p.Leu452Pro	borderline	no	no	no	no	S
ITM2011-00579	ERR4553852	M	45	01/11/2010	3+	none	none	no	lineage1	lineage1.2.2	lineage1.2	rpoB_p.Asp435Tyr	borderline	rpoC	yes	no	no	S
ITM2011-00618	ERR4553854	M	49	22/12/2010	2+	none	none	no	lineage1	lineage1.1.3.1	lineage1.1	rpoB_p.His445Tyr	common	no	no	no	no	S
ITM2011-00957	ERR4553858	M	49	09/02/2011	1+	none	none	no	lineage1	lineage1.1.3.1	lineage1.1	rpoB_p.Gln432Lys	common	no	no	no	no	S
ITM2011-01158	ERR4553862	M	36	09/03/2011	2+	none	none	no	lineage1	lineage1.2.2.2	lineage1.2	rpoB_p.Ile491Phe	borderline	no	no	no	no	S
ITM2011-01200	ERR4553865	M	31	16/03/2011	3+	none	none	no	lineage3	lineage3	lineage3	rpoB_p.Ser450Leu	common	no	no	no	no	S
ITM2011-01211	ERR4553867	M	30	23/03/2011	3+	none	none	no	lineage2	lineage2.2.1	lineage2.2	rpoB_p.Ser450Leu	common	no	no	no	no	S
ITM2011-01332	ERR4553871	M	32	31/03/2011	3+	none	none	no	lineage1	lineage1.1.2	lineage1.1	rpoB_p.Asp435Tyr	borderline	no	no	no	no	S
ITM2011-01472	ERR4553876	M	16	26/04/2011	1+	none	none	no	lineage2	lineage2.2.1	lineage2.2	rpoB_p.Ser450Trp	common	no	no	no	no	S
ITM2011-01507	ERR4553881	M	35	10/05/2011	2+	none	none	no	lineage1	lineage1.1.3.1	lineage1.1	rpoB_p.Ser450Leu	common	no	no	no	no	S

Supplementary Table 2. (Combinations of) *rpoB* mutations found and their classification as "associated with resistance", "associated with resistance interim", or "borderline" in the "WHO Catalogue of mutations in *Mycobacterium tuberculosis* complex and their association with drug resistance", or as "not listed in the WHO catalogue but located within the RRDR", or "not listed in the WHO catalogue and not located within the RRDR (unknown association)" [15]. *rpoB* mutations were further grouped as "common", "low-confidence" or "borderline as described in the materials and methods section. Isolates within a transmission cluster had a maximum difference of 5 SNPs with at least one other isolate in the cluster and had the same *rpoB* mutation.

Type of rpoB mutation	Total (n)	In a cluster (n)	Unique (n)
**Common**			
1296_1297insTTC	2	0	2
1296_1297insTTC, 1299_1300insTTG	1	0	1
Val170Phe	5	0	5
Val170Phe, His445Tyr	1	0	1
Gln432Lys	3	0	3
Gln432Pro	2	0	2
Asp435Val	16	4	12
Ser441Leu	1	0	1
His445Arg	4	0	4
His445Asp	24	2	22
His445Cys	2	0	2
His445Tyr	30	7	23
Ser450Leu	210	92	118
Ser450Leu, 1306_1308del	1	1	0
Ser450Leu, Ala286Val	1	0	1
Ser450Leu, Ile480Val	1	0	1
Ser450Leu, Glu761Asp	1	1	0
Ser450Trp	17	4	13
**Low-confidence**			
Thr427Pro, His445Tyr	3	3	0
Leu430Arg, Ser450Val	1	0	1
Ser431Arg, Asp435Gly, Asn437Asp	1	0	1
Gln432Arg	1	0	1
Met434Thr, Asp435Gly	2	2	0
Asp435Gly, His445Asp	1	0	1
His445Gln, Ser450Trp	1	0	1
His445Gly	1	0	1
His445Pro	1	0	1
Ser450Gly	1	0	1
**Borderline**			
Leu430Pro	8	4	4
Leu430Pro, Ser493Leu	1	0	1
Asp435Tyr	17	7	10
Asp435Tyr, Ser431Asn	1	1	0
Asp435Tyr, Met434Arg	1	1	0
His445Asn	6	5	1
His445Asn, Asp435Glu	1	0	1
His445Leu	4	0	4
His445Leu, Ser428Thr	3	3	0
Leu452Pro	11	0	11
Ile491Phe	6	0	6

Supplementary Figure 1. Flowchart of sample selection.

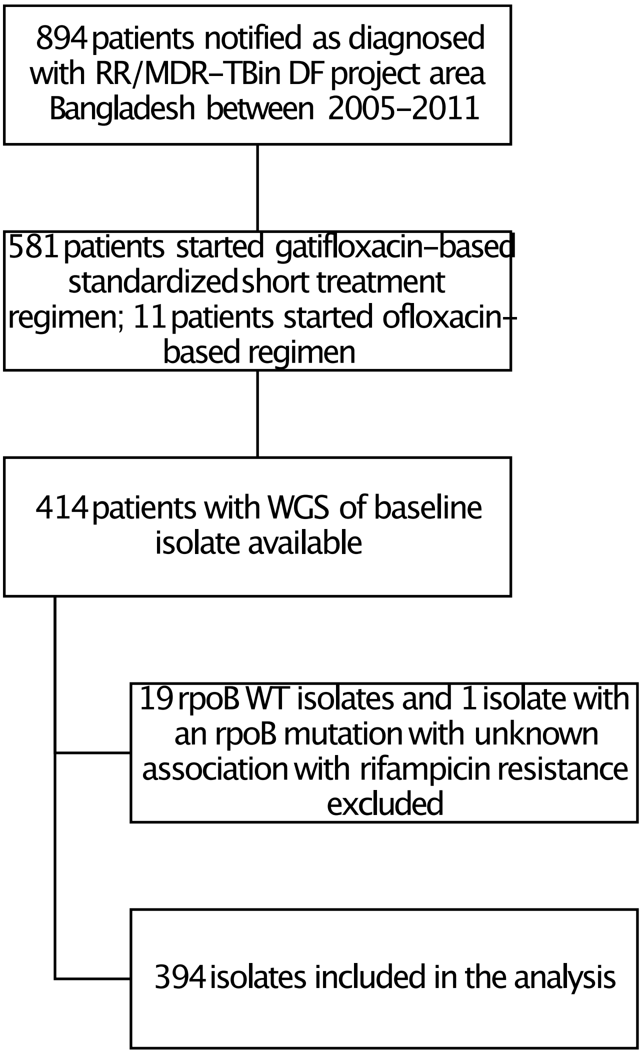

Supplementary Figure 2. SNP distances within transmission clusters. Samples were clustered using a 5 SNP clustering approach with single linkage criterion between members in a cluster. The minimum, maximum and average SNP distance between samples in each cluster are shown here.

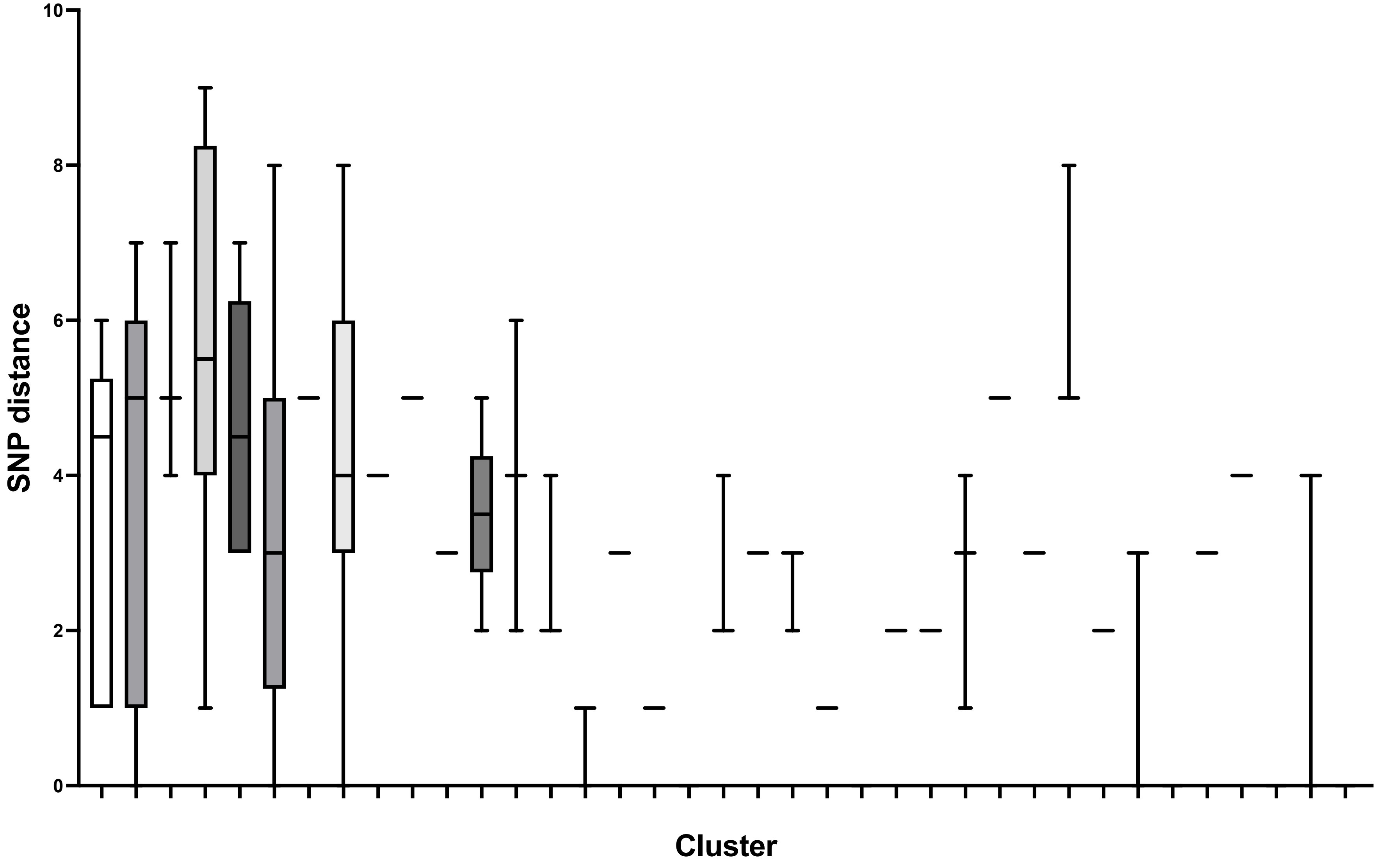

Supplement: Supplementary material 1 [file mgen-9-1109-s001.pdf]
